# Chronic lung diseases are associated with gene expression programs favoring SARS-CoV-2 entry and severity

**DOI:** 10.1038/s41467-021-24467-0

**Published:** 2021-07-14

**Authors:** Linh T. Bui, Nichelle I. Winters, Mei-I Chung, Chitra Joseph, Austin J. Gutierrez, Arun C. Habermann, Taylor S. Adams, Jonas C. Schupp, Sergio Poli, Lance M. Peter, Chase J. Taylor, Jessica B. Blackburn, Bradley W. Richmond, Andrew G. Nicholson, Doris Rassl, William A. Wallace, Ivan O. Rosas, R. Gisli Jenkins, Naftali Kaminski, Jonathan A. Kropski, Nicholas E. Banovich, Human Cell Atlas Lung Biological Network, Alexander V. Misharin, Alexander M. Tsankov, Avrum Spira, Pascal Barbry, Alvis Brazma, Christos Samakovlis, Douglas P. Shepherd, Emma L. Rawlins, Fabian J. Theis, Jennifer Griffonnet, Haeock Lee, Herbert B. Schiller, Paul Hofman, Joseph E. Powell, Joachim L. Schultze, Jeffrey Whitsett, Jiyeon Choi, Joakim Lundeberg, Naftali Kaminski, Jonathan A. Kropski, Nicholas E. Banovich, Jose Ordovas-Montanes, Jayaraj Rajagopal, Kerstin B. Meyer, Mark A. Krasnow, Kourosh Saeb‐Parsy, Kun Zhang, Robert Lafyatis, Sylvie Leroy, Muzlifah Haniffa, Martijn C. Nawijn, Marko Z. Nikolić, Maarten van den Berge, Malte Kuhnemund, Charles-Hugo Marquette, Michael Von Papen, Oliver Eickelberg, Orit Rosenblatt-Rosen, Paul A. Reyfman, Dana Pe’er, Peter Horvath, Purushothama Rao Tata, Aviv Regev, Mauricio Rojas, Max A. Seibold, Alex K. Shalek, Jason R. Spence, Sarah A. Teichmann, Stephen Quake, Thu Elizabeth Duong, Tommaso Biancalani, Tushar Desai, Xin Sun, Laure Emmanuelle Zaragosi

**Affiliations:** 1 Translational Genomics Research Institute, Phoenix, AZ USA; 2 Division of Allergy, Pulmonary and Critical Care Medicine, Department of Medicine, Vanderbilt University Medical Center, Nashville, TN USA; 3 Respiratory Medicine NIHR Biomedical Research Centre, University of Nottingham, Nottingham, UK; 4 Section of Pulmonary, Critical Care and Sleep Medicine, Yale School of Medicine, New Haven, CT USA; 5 Division of Pulmonary and Critical Care Medicine, Brigham and Women’s Hospital, Harvard Medical School, Boston, MA USA; 6 Department of Veterans Affairs Medical Center, Nashville, TN USA; 7 National Heart and Lung Institute, Imperial College, London, UK; 8 Department of Histopathology, Royal Brompton and Harefield NHS Foundation Trust, London, UK; 9 Pathology Research, Royal Papworth Hospital NHS Foundation Trust, Cambridge, UK; 10 Department of Pathology, Royal Infirmary of Edinburgh, Edinburgh, UK; 11 Division of Pathology, Edinburgh University Medical School, Edinburgh, UK; 12 Pulmonary, Critical Care and Sleep Medicine, Department of Medicine, Baylor College of Medicine, Houston, TX USA; 13 Department of Cell and Developmental Biology, Vanderbilt University, Nashville, TN USA; 14 Division of Pulmonary and Critical Care Medicine, Northwestern University, Chicago, IL USA; 15 Genetics and Genomic Sciences, Icahn School of Medicine at Mount Sinai, New York, NY USA; 16 Department of Medicine, Boston University School of Medicine, Boston, MA USA; 17 Johnson & Johnson Innovation, Cambridge, MA USA; 18 Université Côte d’Azur, CNRS, IPMC, Sophia-Antipolis, France; 19 European Molecular Biology Laboratory, European Bioinformatics Institute (EMBL-EBI), Wellcome Trust Genome Campus, Hinxton, Cambridge, UK; 20 SciLifeLab, Department of Molecular Biosciences, Stockholm University, Stockholm, Sweden; 21 Cardiopulmonary Institute, Justus Liebig University, Giessen, Germany; 22 Center for Biological Physics and Department of Physics, Arizona State University, Tempe, AZ USA; 23 Wellcome Trust/CRUK Gurdon Institute, Cambridge, UK; 24 Department Physiology, Development and Neuroscience, University of Cambridge, Cambridge, UK; 25 Institute of Computational Biology, Helmholtz Zentrum München, Oberschleißheim, Germany; 26 Departments of Mathematics and Life Sciences, Technical University Munich, Munich, Germany; 27 Pneumology Department, Nice University-Affiliated Hospital, Nice, France; 28 Department of Biomedicine and Health Sciences, The Catholic University of Korea, Seoul, Korea; 29 Comprehensive Pneumology Center (CPC)/Institute of Lung Biology and Disease (ILBD), Helmholtz Zentrum München, Oberschleißheim, Germany; 30 Member of the German Center for Lung Research (DZL), Munich, Germany; 31 Laboratory of Clinical and Experimental Pathology, Pasteur Hospital, University Côte d’Azur, Nice, France; 32 Hospital‐Related Biobank, Pasteur Hospital, University Côte d’Azur, Nice, France; 33 FHU OncoAge, Pasteur Hospital, BP69, Nice cedex 01, France; 34 Garvan-Weizmann Centre for Cellular Genomics, Garvan Institute of Medical Research, Sydney, NSW Australia; 35 UNSW Cellular Genomics Futures Institute, University of New South Wales, Sydney, NSW Australia; 36 Department for Genomics & Immunoregulation, LIMES-Institute, University of Bonn, Bonn, Germany; 37 PRECISE Platform for Single Cell Genomics & Epigenomics, Germany Center for Neurodegenerative Diseases and University of Bonn, Bonn, Germany; 38 Cincinnati Children’s Hospital Medical Center, Cincinnati, OH USA; 39 Division of Cancer Epidemiology and Genetics, National Cancer Institute, Bethesda, MD USA; 40 SciLifeLab, Department of Gene Technology, KTH Royal Institute of Technology, Stockholm, Sweden; 41 Broad Institute of MIT and Harvard, Cambridge, MA USA; 42 Division of Gastroenterology, Hepatology, and Nutrition, Boston Children’s Hospital, Boston, MA USA; 43 Harvard Stem Cell Institute, Cambridge, MA USA; 44 Program in Immunology, Harvard Medical School, Boston, MA USA; 45 Cellular Genetics Programme, Wellcome Sanger Institute, Wellcome Genome Campus, Hinxton, Cambridge, UK; 46 Department of Biochemistry and Wall Center for Pulmonary Vascular Disease, Stanford University, Stanford, CA USA; 47 Department of Surgery, University of Cambridge and NIHR Cambridge Biomedical Research Centre, Cambridge, UK; 48 Department of Bioengineering, UCSD, La Jolla, CA USA; 49 Division of Rheumatology, Department of Medicine, University of Pittsburgh Medical Center, Pittsburgh, PA USA; 50 Department of Pulmonary Medicine and Allergology, Université Côte d’Azur, CHU de Nice, FHU OncoAge, Nice, France; 51 CNRS UMR 7275—Institut de Pharmacologie Moléculaire et Cellulaire, Sophia Antipolis, France; 52 Wellcome Sanger Institute, Wellcome Genome Campus, Hinxton, Cambridge, UK; 53 Biosciences Institute, Faculty of Medical Sciences, Newcastle University, Newcastle upon Tyne, UK; 54 Department of Dermatology and NIHR Newcastle Biomedical Research Centre, Newcastle Hospitals NHS Foundation Trust, Newcastle upon Tyne, UK; 55 Department of Pathology and Medical Biology, University of Groningen, GRIAC Research Institute, University Medical Center Groningen, Groningen, the Netherlands; 56 UCL Respiratory, Division of Medicine, University College London, London, UK; 57 Department of Pulmonary Diseases, University Medical Center Groningen, Groningen, the Netherlands; 58 Groningen Research Institute for Asthma and COPD, University of Groningen, Groningen, the Netherlands; 59 Cartana AB, Stockholm, Sweden; 60 Team 4, IRCAN, FHU OncoAge, University Côte d’Azur, CNRS, INSERM, Nice CEDEX 02, France; 61 Department of Pneumology and Oncology, CHU Nice, FHU OncoAge, University Côte d’Azur, Nice, France; 62 Comma Soft AG, Bonn, Germany; 63 Department of Medicine, University of Pittsburgh, Pittsburgh, PA USA; 64 Klarman Cell Observatory, Broad Institute of MIT and Harvard, Cambridge, MA USA; 65 Division of Pulmonary and Critical Care Medicine, Northwestern University Feinberg School of Medicine, Chicago, IL USA; 66 Computational and Systems Biology Program, Sloan Kettering Institute, Memorial Sloan Kettering Cancer Center, New York, NY USA; 67 Biological Research Centre of the Hungarian Academy of Sciences, Szeged, Hungary; 68 Institute for Molecular Medicine Finland (FIMM), University of Helsinki, Tukholmankatu, Helsinki, Finland; 69 Department of Cell Biology, Regeneration Next Initiative, Duke University School of Medicine, Durham, NC USA; 70 Department of Biology, Howard Hughes Medical Institute, MIT, Cambridge, MA USA; 71 Genentech, South San Francisco, CA USA; 72 Division of Pulmonary, Allergy and Critical Care Medicine, University of Pittsburgh, Pittsburgh, PA USA; 73 Department of Pediatrics; Center for Genes, Environment, and Health, National Jewish Health, Denver, CO USA; 74 Ragon Institute of MGH, MIT, and Harvard, Cambridge, MA USA; 75 Institute for Medical Engineering and Science (IMES), Koch Institute for Integrative Cancer Research, Cambridge, MA USA; 76 Department of Chemistry, Massachusetts Institute of Technology, Cambridge, MA USA; 77 Department of Internal Medicine, Gastroenterology, University of Michigan Medical School, Ann Arbor, MI USA; 78 Department of Cell and Developmental Biology, University of Michigan Medical School, Ann Arbor, MI USA; 79 Department of Biomedical Engineering, University of Michigan College of Engineering, Ann Arbor, MI USA; 80 Department of Physics/Cavendish Laboratory, University of Cambridge, JJ Thompson Ave, Cambridge, UK; 81 Chan Zuckerberg Biohub, San Franscisco, CA USA; 82 Department of Pediatrics, Respiratory Medicine, University of California, San Diego, CA USA; 83 Department of Medicine and Institute for Stem Cell Biology and Regenerative Medicine, Stanford University School of Medicine, Stanford, CA USA; 84 Department of Pediatrics, University of California, San Diego, San Diego, CA USA; 85 Laboratory of Genetics, University of Wisconsin–Madison, Madison, WI USA; 86 Université Côte d’Azur, CNRS, IPMC, Sophia-Antipolis, France

**Keywords:** Computational biology and bioinformatics, Viral infection, Inflammation, Viral infection, Immunopathogenesis

## Abstract

Patients with chronic lung disease (CLD) have an increased risk for severe coronavirus disease-19 (COVID-19) and poor outcomes. Here, we analyze the transcriptomes of 611,398 single cells isolated from healthy and CLD lungs to identify molecular characteristics of lung cells that may account for worse COVID-19 outcomes in patients with chronic lung diseases. We observe a similar cellular distribution and relative expression of SARS-CoV-2 entry factors in control and CLD lungs. CLD AT2 cells express higher levels of genes linked directly to the efficiency of viral replication and the innate immune response. Additionally, we identify basal differences in inflammatory gene expression programs that highlight how CLD alters the inflammatory microenvironment encountered upon viral exposure to the peripheral lung. Our study indicates that CLD is accompanied by changes in cell-type-specific gene expression programs that prime the lung epithelium for and influence the innate and adaptive immune responses to SARS-CoV-2 infection.

## Abstract (web summary)

Patients with chronic lung disease (CLD) have an increased risk for severe coronavirus disease-19 and poor outcomes. Here the authors compare the transcriptomes of single cells isolated from healthy and CLD lungs to identify molecular characteristics of lung cells that may account for worse COVID-19 outcomes in these patients.

## Introduction

In December 2019, a respiratory disease associated with a novel coronavirus emerged in Wuhan, China^1–3^. The syndrome, now called COVID-19, was caused by severe acute respiratory syndrome coronavirus 2 (SARS-CoV-2) and has since rapidly spread worldwide^4^. As of May 18, 2021, a total of over 163 million confirmed COVID-19 cases and more than 3.3 million deaths have been reported around the globe^5^.

The clinical manifestations of SARS-CoV-2 infection range from asymptomatic to fulminant cases of acute respiratory distress syndrome (ARDS) and life-threatening multi-system organ failure. Development of ARDS in patients with SARS-CoV-2 dramatically increases the risk of ICU admission and death^6–12^. Risk factors for severe SARS-CoV-2 include age, smoking status, ethnicity and male sex^13–15^. Baseline comorbidities including hypertension, diabetes and obesity, increase SARS-CoV-2 susceptibility and severity^1,10,16–19^. In addition, chronic lung disease (CLD) has been identified as a risk factor for hospitalization and mortality in patients with COVID-19^20–27^. Patients with chronic obstructive pulmonary disease (COPD) and interstitial lung disease (ILDs), especially Idiopathic Pulmonary Fibrosis (IPF), have a significantly higher COVID-19 mortality rate compared to patients without chronic lung disease^28^. However, the molecular mechanisms underlying the increased risk of SARS-CoV-2 severity and mortality in patients with pre-existing lung diseases are not well understood.

In this work, we performed an integrated analysis of four lung single cell RNA-sequencing (scRNA-seq) datasets^29–32^ in addition to unpublished data, together including 78 control and 132 CLD samples (*n* = 31 COPD, 82 IPF and 19 other ILDs), to investigate the molecular basis of SARS-CoV-2 severity and mortality risk in CLD patients. We found that CLD is associated with baseline changes in cell-type specific expression of genes related to viral replication and the immune response, as well as evidence of immune exhaustion and altered inflammatory gene expression. Together, these data provide a molecular framework underlying the increased risk of SARS-CoV-2 severity and poor outcomes in patients with certain pre-existing CLD.

## Results

### Integrated analysis of lung single cell RNA sequencing datasets

To determine why COVID-19 patients with CLD have a higher risk of severe infection and poorer outcomes, we performed an integrated analysis on four published scRNA-seq lung datasets: Northwestern (biopsy deemed representative of explanted lung), Pittsburgh and VUMC/TGen (biopsy of apical and basal region of explanted lung) and Yale/BWH (longitudinal biopsy through explanted lung) (Supplementary Table 1a), in addition to previously unpublished samples (VUMC/TGen). We analyzed the transcriptomes from 611,398 single cells derived from healthy donors (78 samples), COPD (31 samples), IPF (82 samples) and Non-IPF ILD (Other ILD, 19 samples) (Supplementary Table 1b, 2). Using published cell type specific markers^31,32^, we identified 32 distinct cell types in the dataset (Supplementary Fig. 1). Overall, we observed similar cell type proportions between the different datasets and diagnosis groups (Supplementary Table 3, Supplementary Fig. 2), with the exception of high AT2 cell numbers in the Northwestern dataset, as expected due to the protocol favoring isolation of AT2 and macrophages (personal communication). Despite the variation in sample collection and processing at different research institutes, the similarity in cell type composition per dataset indicated the compatibility of samples and that no major sampling bias would confound an integrated analysis.

### Expression profile of SARS-CoV-2 associated receptors and factors in the diseased lung

**Fig. 1:**
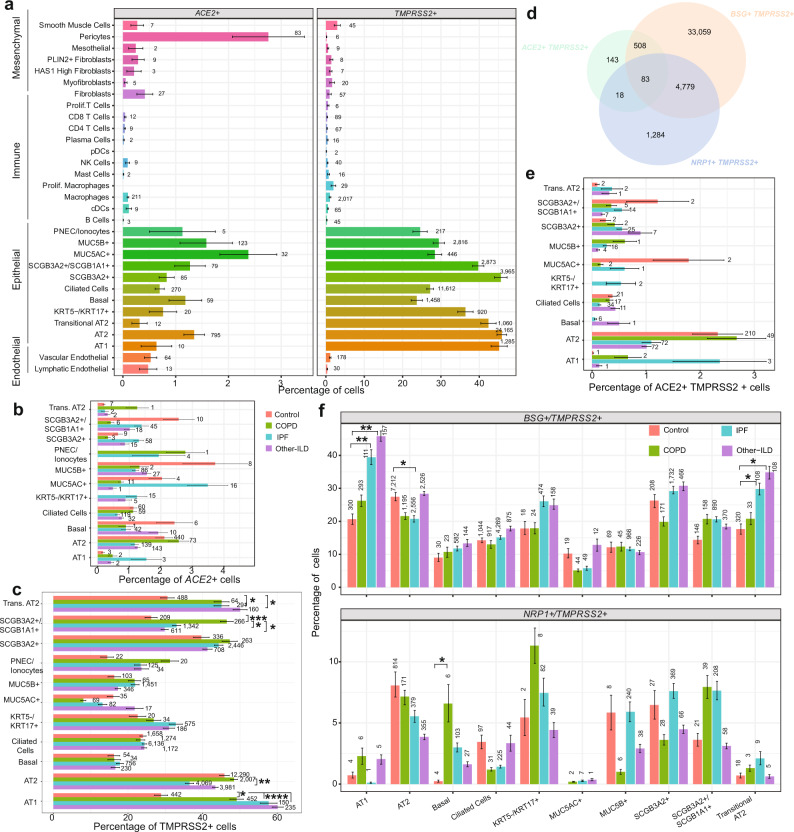
Percentage of cells expressing SARS-CoV-2 receptor genes in lung cell types in different diagnosis subgroups. **a** Percentage of cells expressing *ACE2* and *TMPRSS2* in all cell types. Numbers are the total number of *ACE2* + or *TMPRSS2* + cells in each cell type in the dataset. **b**, **c** Percentage of cells expressing *ACE2* (**b**) and *TMPRSS2* (**c**) in each diagnosis group in the epithelial cell types. **d** Venn diagram shows overlapping of cells co-expressing the proposed receptors (*ACE2*, *BSG* and *NRP1*) and the protease *TMPRSS2*. **e**, **f** Percentage of cells co-expressing receptors and *TMPRSS2* split by cell type and diagnosis group. Plots were generated with mean values of percentage of cells per individual samples, and data are presented as mean values ± SEM. Significant differences between diagnosis groups were calculated using Tukey_HSD test, *p* value < 0.05: *p value < 0.01: **p value < 0.001: ***p value < 0.0001: ****.

SARS-CoV-2 utilizes the host *ACE2*, and other putative factors such as *BSG, NRP1* and *HSPA5*, as entry receptors and *TMPRSS2*, *CTSL* or *FURIN* as priming proteases to facilitate cellular entry^33–40^. Consistent with prior reports analyzing normal lung tissue^33,34,41^, *ACE2* and *TMPRSS2* are expressed predominantly in epithelial cell types (Fig. 1a), while other putative SARS-CoV-2 entry receptors (*BSG*, *NRP1*, *HSPA5*) and priming proteases (*CTSL*, *FURIN*) have substantially more widespread expression in nearly all cell types (Supplementary Fig. 3). The total number and proportion of *ACE2* + cells are highest in pericytes, type 2 alveolar cells (AT2) and secretory cells, while *TMPRSS2* is widely expressed in all epithelial cell types. There were no significant differences in the proportion of *ACE*+ cells in any cell-type in CLD versus control groups (Fig. 1b). The proportion of *TMPRSS2* + AT2 cells is decreased in IPF lungs while *TMPRSS2* + AT1 and Transitional AT2 cells are higher in all CLD samples; and *TMPRSS2* + *SCGB3A2* +/*SCGB1A*+ club cells are in significantly higher numbers in COPD patients compared to controls (Fig. 1c). The putative entry factor *NRP1* is expressed in more pDCs, myofibroblasts and HAS1 high fibroblasts in CLD samples compared to control (Supplementary Fig. 3).

Next, we compared the number of double positive cells, i.e., cells co-expressing a receptor and priming protease, in control and CLD samples. A notable fraction of cells co-expresses all established and putative entry receptors (*ACE2*, *BSG*, *NRP1*, *HSPA5*) and proteases (*TMPRSS2*, *CTSL*, *FURIN*); AT2 cells comprised nearly half of all such cells (43.3%) (Fig. 1d, Supplementary Fig. 4). While the percentage of cells co-expressing*ACE2* and priming proteases (*TMPRSS2*, *CTSL*, *FURIN*) was similar across disease subtypes, there was a significantly higher number of cells co-expressing*ACE2* and *FURIN* in the COPD AT2 and Transitional AT2 cells (Fig. 1e, Supplementary Fig. 4). We detected significant differences in the number of cells co-expressing*BSG*, *NRP1*, *HSPA5* with a priming protease in CLD samples in multiple cell types (Fig. 1f, Supplementary Fig. 4).

**Fig. 2:**
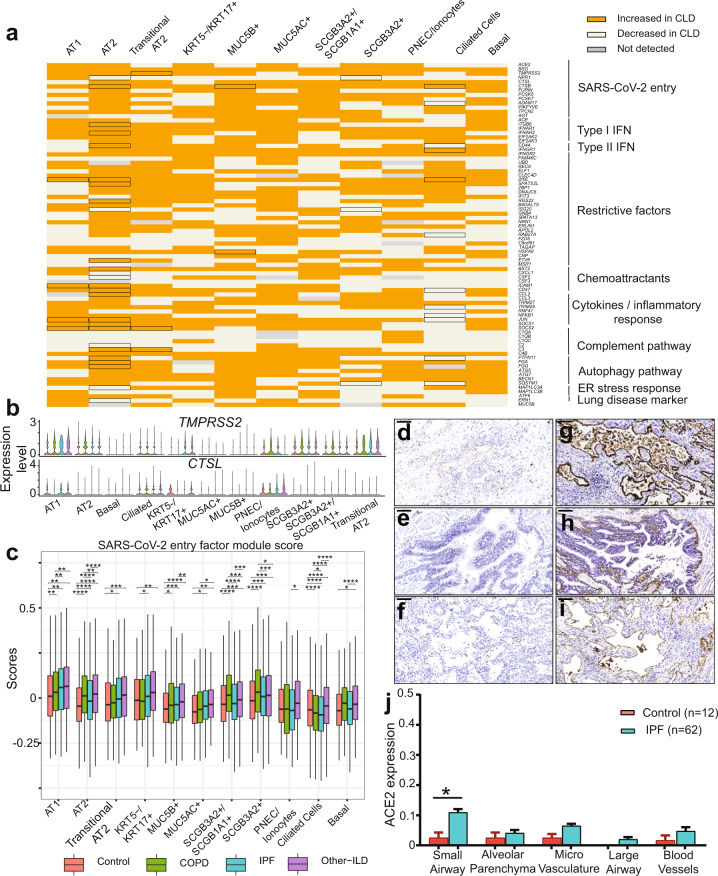
Expression profile of SARS-CoV-2 mediators and response genes in the epithelial cell population. **a** Binary heatmap representing a manually curated list of genes associated with SARS-CoV-2. Orange elements indicate genes with increased expression and white elements indicate genes with decreased expression in CLD samples; Not detected: gene expression was not detected in either of the two tested populations (CLD vs. Control). Differential expressed genes (DEGs) between CLD and control samples (FDR ≤ 0.1) are outlined in black. **b** Violin plot depicts gene expression level in CLD and control of the two SARS-CoV-2 proteases *TMPRSS2* and *CTSL*. **c** SARS-CoV-2 entry module score in different cell types, SARS-CoV-2 mediators included *ACE2, BSG (CD147), NPR1, HSPA5 (GRP78), TMPRSS2, CTSL, ADAM17, FURIN*. The outliers were removed in this plot, please see Supplementary Fig. 8a with outliers included. Boxes: interquartile range, lower and upper hinges correspond to the first and third quantiles, upper and lower whisker extends from the hinge to the largest values or smallest values of 1.5 x interquartile range; *p value < 0.05, **p value < 0.01, ***p value < 0.001, ****p-value < 0.0001, Tukey_HSD post-hoc test. ACE2 and ITGB6 protein expression in IPF lung sections. IPF lung sections stained for ACE2: **d** small airway, **e** large airway and **f** lung parenchyma. IPF lung sections stained for αvβ6: **g** small airway, **h** large airway and **i** lung parenchyma. **j** Semi-quantitative evaluation of ACE2 scoring among control (*n* = 12 for each tissue) and IPF (*n* = 62 for each tissue) sections (both the percentage of staining and staining intensity of ACE2 expression; 0-Negative; 1–0–⩽10%; 2-11–⩽25%; 3-⩽26%), data are presented as mean values ± SEM. Significant differences between IPF and control were calculated using Tukey HSD test, *p* value < 0.05 *. Scale bar = 100 µm. For **d**–**j**: a total of 12 normal lung samples and 62 IPF samples were used.

To examine whether CLD patients express higher levels of SARS-CoV-2 receptors and priming proteases, we performed differential expression analysis of those genes in the CLD versus control samples. The two major SARS-CoV-2 cellular entry factors, *ACE2* and *TMPRSS2*, have similar expression profiles in the disease and control samples. *ACE2* expression is relatively low in all cell types and there were no significant differences in *ACE2* expression in CLD groups compared to control (Supplementary Fig. 5). The putative alternative receptor *NRP1*, recently confirmed as another host entry factor for SARS-CoV-2^35^, is slightly up-regulated in the COPD macrophages, but down-regulated in both IPF and Other-ILD macrophages (Supplementary Fig. 6). *TMPRSS2* expression is high in AT1, AT2, Transitional AT2, PNEC/ionocytes and club cells (Fig. 2b, Supplementary Fig. 5) and is slightly upregulated in the AT2 COPD samples (log2FC = 0.28, *q* value 0.04) (Supplementary Dataset 3), in contrast to a recent publication demonstrating decreased *TMPRSS2* expression in severe COPD^42^. Two alternative priming proteases (*CTSL* and *FURIN*) are expressed at low level and show no significant differences in expression between control and disease samples (Fig. 2b, Supplementary Fig. 5). However, the SARS-CoV-2 entry gene score (calculated on the average expression levels of all SARS-CoV-2 entry factors over a random control gene set) is significantly increased in the CLD samples in many epithelial cell types, including AT1, AT2, Basal, Club cells, and *KRT5-/KRT17* + cells, an ECM-producing epithelial cell type enriched in the fibrotic lung^31,32^ (Fig. 2c, Supplementary Fig. 8a, b). Together, these data suggest CLD is associated with modest changes in expression of established SARS-CoV-2 entry factors, and alternative mechanisms are likely additionally responsible for observed differences in outcome severity.

### Dysregulation of viral infection and innate immune response genes in disease epithelial cells

Given the relatively modest differences in SARS-CoV-2 entry factors in epithelial cells between CLD and control lungs (Fig. 2b, Supplementary Figs. 5, 6), we hypothesized that rather than greatly increased cellular susceptibility to SARS-CoV-2 infection, patients with CLD are predisposed to severe lung injury due to underlying differences in epithelial gene expression in key pathways mediating the antiviral response. Focusing on the epithelial cell population (a total of 143,114 cells), we selectively examined genes that have been demonstrated in the SARS, MERS and rapidly expanding COVID-19 literature to impact viral pathogenesis. We noted that many of these genes are significantly dysregulated (Bonferroni adjusted p-value, FDR, ≤ 0.1) in several epithelial cell types (Fig. 2a). Included are genes thought to directly impact viral replication (*TMPRSS2, NPR1, CTSB*), interferon stimulated genes (ISGs) thought to be involved in restricting viral entry and replication (*LY6E, SPATS2L*)^43^, and key regulators of the host viral response including cytokine and inflammatory response genes (IFN type I and type II receptors, *SOCS1*/*2*, *CCL2, CD47*) (Fig. 2a, Supplementary Fig. 8c, d). In addition, the complement pathway gene *C3*, an important component of the innate immune response and previously found to be elevated in SARS patients^44^, and autophagy (*FGG, FGA, PTPN11*) genes are also significantly dysregulated in many CLD epithelial cells; these pathways are important for propagating viral infection and the host response^45–47^. Among the epithelial cell types, AT2 cells have the largest number of significantly dysregulated genes in CLD compared to control samples (Fig. 2a). These data suggest that there are basal differences in the expression profiles of genes regulating viral infection and the immune response in diseased epithelial cells, in particular in AT2 cells, and that this epithelial “priming” may contribute to COVID-19 severity and poor outcomes.

### Elevated ACE2 protein expression level in the small airways in IPF lungs

**Fig. 3:**
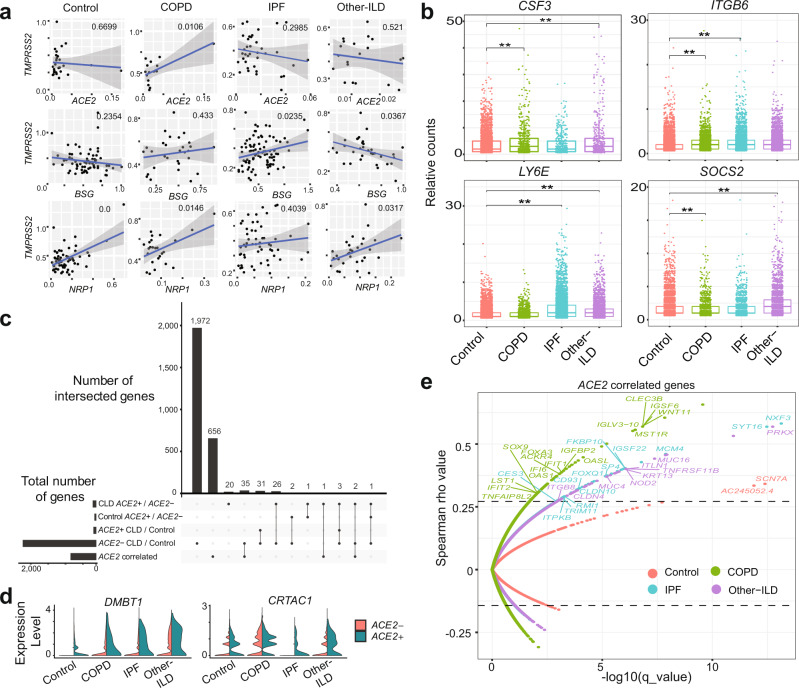
CLD AT2 cells exhibit baseline differences in gene expression profile coping with viral infection. **a** Significant gene expression correlation in AT2 cells between *TMPRSS2* and *ACE2*, *BSG* (*CD147*) and *NPR1* in COPD and IPF samples, each dot represents the average expression level of the genes of interest per sample, pairwise gene correlation analysis was done using a fitting linear model and *p* value was calculated using Anova. **b** Boxplot shows differences in gene expression of selected SARS-CoV-2 response genes in the AT2 cell types among different diagnosis groups, Boxes: interquartile range, lower and upper hinges correspond to the first and third quantiles, upper and lower whisker extends from the hinge to the largest values or smallest values of 1.5 × interquartile range; **p value-adj ≤ 0.05 (negative binomial test, corrected for Age, Ethnicity, Smoking_status and Dataset). **c** Upset plot shows shared differential expression genes (DEGs) between different comparisons: *ACE2−* CLD vs. Control, *ACE2* + CLD vs. Control, CLD *ACE2* + vs. *ACE2-*, Control *ACE2* + vs. *ACE2-* and *ACE2* correlated genes in the AT2 cells. **d** Upregulation of two genes uniquely differentially expressed in the CLD *ACE2* + vs. *ACE2−*. **e** Spearman gene correlation analysis identified genes correlated with *ACE2* expression in AT2 *ACE2* + cells in different diagnosis groups, p-value was adjusted using Benjamini-Hochberg corrections, dashed lines indicate the 99th percentile of Spearman rho values.

To further study the expression of the major SARS-CoV-2 entry factor *ACE2* in the fibrotic lungs, we examined protein levels of ACE2 in different lung regions using the anti-ACE2 ab108252 antibody (Supplementary Fig. 9a). In agreement with the transcript quantification above and previous immunohistology analysis^48^, we detected overall low expression level of ACE2 across all tissue types in both IPF (Fig. 2d–f) and control lung sections (Supplementary Fig. 9b, c). Semi-quantitative evaluation of ACE2 expression scoring showed elevated ACE2 expression in all IPF sections compared to control, reaching statistical significance in the IPF small airway sections (Fig. 2j), suggesting that while overall ACE2 expression is low, there is a regional concentration of ACE2 + cells within the distal IPF lung that may promote a more severe localized viral response. Upregulation of the epithelial integrin alpha-V beta-6 (αvβ6) plays an important role in enhanced fibrosis in response to lung injury^49^, and enhances TGFβ activation which can suppress type I interferon responses from alveolar macrophages increasing susceptibility to viral infection^50^. We detected a significant increase of αvβ6 integrin expression in all lung sections isolated from IPF patients (Fig. 2g–i). While there was additional positive staining in the peripheral lung, αvβ6 expression is highest in the AT2 epithelial cells in the IPF samples compared to overall low expression level in the normal lung sections (Supplementary Fig. 9b, c), mirroring the expression data of *ITGB6* described below (Fig. 3b).

### CLD specific *ACE2*+ transcriptional profiles in AT2 cells

In the distal lung, AT2 cells have been proposed to be the primary targets of SARS-CoV-2^34,41,51^ and comprise the initial microenvironment the virus encounters. Thus, we examined the gene expression profile of CLD AT2 cells in more detail. As described above, AT2 cells in all diagnosis subgroups have significantly higher SARS-CoV-2 entry gene scores than control cells (Fig. 2c). In addition, CLD AT2 cells express higher levels of many genes related to viral infection and innate immune responses than any epithelial cell type (Fig. 2a). COPD and Other-ILD, but not IPF, AT2 cells express higher levels of *CSF3*, an important cytokine in the regulation of granulocytes, and the suppressor of cytokine signaling-2 (*SOCS2*) (Fig. 3b). The epithelial integrin *ITGB6*, involved in wound healing and pathogenic fibrosis^52^, is upregulated in COPD and IPF AT2 cells; the ISG lymphocyte antigen 6 complex (*LY6E*), known to restrict SARS-CoV-2 entry^43,53^, is upregulated in the IPF and Other-ILD AT2 cells (Fig. 3b, Supplementary Fig. 8e). Gene correlation analysis showed strong positive correlation between *TMPRSS2* and *ACE2*, *NRP1* in COPD AT2 cells (Fig. 3a, Supplementary Fig. 10). *NRP1* expression is also positively correlated with the protease *FURIN* in the AT2 cells isolated from IPF samples (Supplementary Fig. 10c).

Since *ACE2* is the best-establishedSARS-CoV-2 entry factor, and AT2 cells accounted for 54.63% of all *ACE2* + epithelial cells, we focused on the transcriptional profile of *ACE2* + AT2 cells. All of the 34 differentially expressed genes (FDR ≤ 0.1) in *ACE2* + AT2 cells between CLD and control overlapped with the *ACE2*- cells CLD vs. control analysis (Fig. 3c), suggesting that these genes reflected the disease state and were not related to *ACE2* expression. However, when we performed the same differential expression analysis on *ACE2* + vs. *ACE2-* CLD cells, we identified 20 unique genes that were dysregulated in CLD *ACE2* + cells (Fig. 3c, Supplementary Table 4). Among these 20 genes, the tumor suppressor *DMBT1*, a glycoprotein that has been shown previously to be highly expressed in *ACE2* + AT2 cells^54^ and can bind to SARS-CoV-2 spike proteins^55^, and the cartilage acidic protein 1 (*CRTAC1*), previously known to be downregulated significantly in COVID-19 patients with severe infection^56^, were upregulated in *ACE2* + compared to *ACE2*- AT2 CLD cells (Fig. 3d).

Next, we sought to identify *ACE2* correlated genes in the *ACE2* + AT2 cells in different disease groups; thus, identifying the immediate cellular environment SARS-CoV-2 encounters upon infecting a host. We performed Spearman correlation analysis with Benjamini–Hochberg adjusted *p* values and identified distinct gene profiles significantly correlated with *ACE2* for each disease group (Fig. 3c, e). There were only two *ACE2* correlated genes in the Control samples with a cutoff of 99th percentile Spearman rho values and *q* value less than 0.03, none of those genes are associated with the immune response. In the disease samples, we identified 706 genes (COPD: 330 genes, IPF: 108 genes and Other-ILD: 268 genes) with significant correlation to *ACE2* (99th percentile rho values, *q* value less than 0.03) (Supplementary Dataset 4). *ACE2* correlated genes are involved in various cellular processes, including viral processes (Supplementary Table 5). Many *ACE2-*correlated genes in the disease samples are associated with the innate and antiviral immune response. In the COPD samples, genes with strong correlation coefficients with *ACE2* include several interferon-induced genes (*IFI6, IFIT1, IFIT2*), a modulator of innate immune function (*OAS1*), the chemokine receptor *ACKR4*, a gene associated with West Nile viral infection (*OASL*), and the ECM regulated transcription factor *SOX9*. In the IPF samples, *ACE2* expression is strongly correlated with the nuclear factor *NXF3*, the transcription factor *SP4*, the antiviral factor *TRIM11*, and the Forkhead Box Q1 (*FOXQ1*). In other ILD diseases (non-IPF related), the integrin *ITGB8*, a member of the TNF receptor family (*TNFRSF11B*), an important component of the immune response system (*NOD2*) and an innate immune pathway component (*ITLN1*) are among the genes with high correlation with *ACE2*. The transcription factor *FOXQ1* was identified among the 20 unique transcription factors specific for SARS-CoV-2 in a recent in silico study^57^, while *OAS1* was among the top 50 genes with a significant correlation coefficient with *ACE2* in a previous study^33^. The presence of immune-associated genes in these gene correlation profiles suggests that in patients with CLD, *ACE2* + AT2 cells are conditioned and primed to express these genes to cope with viral infection.

### Baseline differences in inflammatory response programs in chronic lung disease

**Fig. 4:**
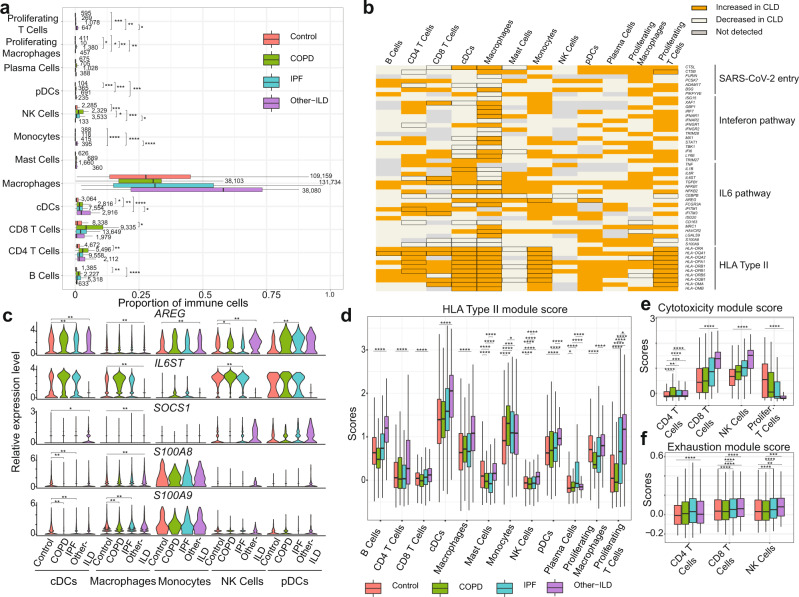
Analysis of SARS-CoV-2 candidate immune response genes in immune cells. **a** Quantification of cell types as a percent of all immune cells in control and diseased lungs, numbers represent the total numbers of the cell type per individual samples, data are presented as mean values ± SEM. **b** Binary heatmap representing a manually curated list of genes associated with SARS-CoV-2. Orange elements indicate genes with increased expression and white elements indicate genes with decreased expression; Not detected: gene expression was not detected in either of the two tested populations (CLD vs. Control); DEGs with FDR ≤ 0.1 are outlined in black. **c** Differential expression analysis for SARS-CoV-2 immune candidate genes in cDCs, Macrophages and Monocytes. *p-adjusted value < 0.1, **p-adjusted value < 0.05, *p*-adjusted value was Bonferroni adjusted from Seurat FindMarkers differential expression analysis using a negative binomial test and corrected for Age, Ethnicity, Smoking_status and Dataset. **d** Compared to the healthy control samples, HLA type II score is higher in all disease groups (especially Other-ILD). In the T cell population, cytotoxicity scores (**e**) and exhaustion scor**e**s (**f**) are higher in the disease samples than in control samples. In **a**, **d**, **e**, and **f**: Boxes: interquartile range, lower and upper hinges correspond to the first and third quantiles, upper and lower whisker extends from the hinge to the largest values or smallest values of 1.5 x interquartile range; Tukey_HSD post-hoc test: *p value < 0.05, **p value < 0.01, ***p value < 0.001, ****p value < 0.0001. See Supplementary Fig. 10 for plots with outliers included for **d**–**f**.

Recent publications have suggested that immune dysregulation, including sustained cytokine production and hyper-inflammation, is associated with SARS-CoV-2 severity^58–61^. We performed an in-depth examination of the immune cell population to determine whether preexisting immune dysregulation in chronic lung disease patients could contribute to SARS-CoV-2 severity and mortality. We analyzed a total of 421,059 cells from 12 immune cell types (Supplementary Table 3, Supplementary Fig. 1) and found significant increases in the proportion of CD4 T Cells, CD8 T Cells, cDCs and NK cells in the disease groups, most notably in COPD samples (Fig. 4a). Similar to Fig. 2a, we examined the expression of SARS-CoV-2 and cellular immune response genes in the CLD immune cells. Several genes related to SARS-CoV-2 entry (*CTSL, CTSB, ADAM17)* and components of the Interferon and IL6 pathways are significantly dysregulated in the CLD Macrophages and cDCs (Fig. 4b). Moreover, many immune cells isolated from CLD samples showed elevated levels of genes in the major histocompatibility complex (MHC) class II genes (HLA type II genes) (Fig. 4b). HLA type II gene module score increased across all disease groups but especially in the Other-ILD samples, compared to controls (Fig. 4d). Type I IFN response (IFNa score) is slightly elevated in the diseased macrophages and pDCs (Supplementary Fig. 11a). IL6-associated tocilizumab responsive genes (IL6 score) are expressed at a higher level in the disease groups IPF and Other-ILD, but lower in the COPD samples (Supplementary Fig. 11b). Previous studies demonstrated elevated exhaustion levels in CD8 T cells in severely affected COVID-19 patients^62,63^. All CLD T cells have higher expression levels of cytotoxicity and exhaustion genes compared to controls (Fig. 4e, f). These perturbations in the T Cell population of CLD lungs may diminish the host immune response to viral infection, leading to a higher risk of severe disease and poor outcomes in response to SARS-CoV-2 infection.

To further investigate differences in immune cell type-specific gene expression profiles, we examined expression levels of genes associated with viral infection in disease versus control samples. Amphiregulin (AREG), a ligand for epidermal growth factor receptor, is known to have essential roles in wound repair and inflammation resolution; furthermore, upregulation of *AREG* is associated with viral infections of the lung^64^. In COVID-19 patients, *AREG* is significantly upregulated in PBMCs^65^, monocytes, CD4 T Cells, NK cells, neutrophils, and DCs^61^, suggesting that upregulation of *AREG* may be an attempt to ameliorate the severe injury induced by SARS-CoV-2 infection. We observed reduced expression of *AREG* in the cDCs and macrophages, but not in the monocytes, in the CLD samples (Fig. 4c, Supplementary Dataset 3, Supplementary Fig. 12). *SOCS1*, a suppressor of cytokine signaling, was shown to reduce the type I IFN antiviral response in bronchial epithelial cells after influenza infection^66,67^. Expression of the S100A8/A9, members of the S100 family, and the IL6 co-receptor*IL6ST* was elevated in COVID-19 patients^68–70^. In our study, *S100A8/A9* expression is lower in the disease samples in cDCs, macrophages and monocytes while *SOCS1* expression is elevated in Other-ILD samples in NK Cells and pDCs (Fig. 4c, Supplementary Dataset 3). *IL6ST* expression level is elevated significantly in COPD and IPF but reduced dramatically in Other-ILD samples in macrophages (log2FC = −2.75, *q* value = 1.63e-61) (Fig. 4c, Supplementary Dataset 3). These basal differences in inflammatory gene expression programs highlight how chronic lung disease alters the inflammatory microenvironment encountered upon viral exposure to the peripheral lung.

## Discussion

**Fig. 5:**
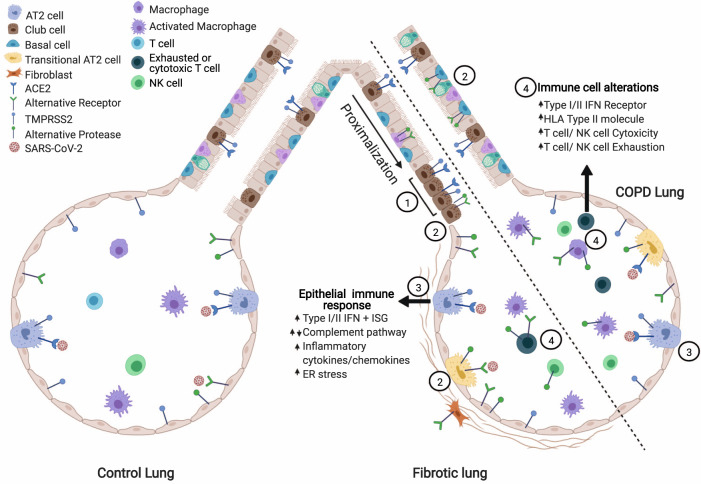
Model of alterations in the diseased lung related to SARS-CoV2 pathogenesis. (**1**) In the IPF lung, there is a proximalization of the distal airway. *ACE2 +* epithelial cells cluster in the small airways though total *ACE2 +* cell numbers are similar to control. (**2**) The viral entry score (accounting for all described putative receptors and proteases) is increased in diseased lungs. (**3**) Diseased epithelial cells have alterations in key SARS-CoV-2 response genes/pathways. (**4**) In the CLD lung, there is increased expression of cytotoxicity and exhaustion genes in immune cell populations and alterations in viral response pathways (interferon, antigen presentation). Figure created in Biorender.com.

The COVID-19 pandemic, caused by the SARS-CoV-2 virus, has affected tens of millions of individuals around the globe in just the first nine months of 2020. Patients with CLD have an increased risk for severe SARS-CoV-2 infection: COPD patients have a five-fold increased risk of severe COVID-19^23,24,71–73^ and ILD patients have up to a four-fold increased odds of death from COVID-19^28,74^. Here, we performed an integrated transcriptomic analysis of scRNA-seq data from healthy and CLD patients to identify potential molecular causative factors determining SARS-CoV-2 severity. To summarize the results (Fig. 5): (1) *ACE2* and *TMPRSS2* are expressed predominantly in epithelial cells and there are no significant differences in the number of *ACE2* + cells in all cell types in disease compared to control samples; (2) a viral entry score including multiple entry factors is increased in cells isolated from diseased lungs; (3) CLD epithelial cells, especially AT2 cells, exhibit pre-existing dysregulation of genes involved in viral infection and the immune response; (4) ACE2 protein levels are elevated in the IPF small airway sections; (5) the CLD *ACE2* + cells differentially express genes related to SARS-CoV-2 infection compared to CLD *ACE2-* AT2 cells; (6) a unique *ACE2* correlated gene profile for each diagnosis group included antiviral and immune regulatory genes; (7) there are baseline differences in the cellular immune population in disease compared to control samples.

Similar to other coronaviruses, SARS-CoV-2 utilizes cellular receptors (*ACE2* and putatively, *BSG, NRP1* and *HSPA5* gene products) and priming proteases (*TMPRSS2, CTSL, FURIN*), for viral entry. These factors are expressed predominantly in the upper and lower airways, with *ACE2* being expressed highly in nasal goblet and ciliated cells and in a subset of AT2 cells and the absorptive enterocytes in the gut^33,34,41,48,51^. We observed a similar expression pattern of *ACE2* in our dataset, with AT2 cells having the highest number *ACE2* + cells. To our knowledge, publications investigating baseline expression of these SARS-CoV-2 entry factors in lung disease have been limited to asthma and COPD with variable results. For example, studies in asthma patients showed elevated expression of *ACE2, TMPRSS2,* and *FURIN* in patients with severe but not mild-moderate asthma^75,76^. Leung et al. performed bulk RNAseq and immunohistochemical staining on bronchial epithelial cells and showed significantly elevated expression levels of *ACE2* and ACE2 protein in the small airways of COPD patients compared to control^77^. Another study on bronchoscopically isolated tissue showed no relationship between disease status (mild to moderate asthma or COPD) on the expression levels of all SARS-CoV-2 entry factors^42^. Our study utilized scRNAseq technology to study gene expression at a very granular level and did not identify increased *ACE2* expression at the single-cell level in CLD, including COPD. However, in the IPF lung, there was a regional concentration of ACE2 + cells in the small airways upon immunohistochemical examination (Fig. 3a, g), similar to the findings of Leung et al.^77^. While the overall frequency of *ACE2* + cells and the ACE2 expression level may be low, changes in the proportional cellular makeup of the diseased lung epithelium may lead to a proportionate increase in *ACE2* + “infectable” cells in the distal lung. Importantly, IPF lungs exhibit abnormal expansion of epithelial cell programs, specifically the presence of more proximal specific cell types in the distal lungs^31,32^. Thus, our data along with previously published studies together suggest that while overall differences in *ACE2* expression and other entry factors may be minimal in CLD, the localization of susceptible cells in the distal lung may promote disease pathogenesis and severity. However, it seems clear that viral entry alone cannot explain the variation in disease severity between patients with and without CLD. In a COVID-19 autopsy study, Dolorey *et al*. found that in these patients who succumbed to complications of infection, SARS-CoV-2 RNA + cells in the lung were largely in the myeloid lineage and did not overlap with entry factor expression^78^. Thus, once the infection has become established and significant cellular injury has taken place, viral entry factor expression may no longer be essential to continued propagation of injury.

A balanced immune response is crucial to viral clearance and avoidance of excessive injury to the host, as evidenced by poor outcomes related both to immunosuppression as well as hyperinflammation in COVID-19 patients^79^. COPD patients with severe COVID-19 had elevated serum levels of various inflammatory cytokines including IL-2R, IL-6, IL-8, IL-10, and TNF-α suggesting there may be global alterations in the immune response^27^. We observed that COPD AT2 cells expressed elevated levels of immune-response related genes (*CSF3, ITGB6, SOCS2*). G-CSF (encoded by *CSF3)* is found at high levels in patients with severe COVID-19 and thought to play a role in the hyperinflammatory syndrome while *SOCS2* is part of a negative feedback system that regulates the response to cytokines^80,81^. *ACE2* correlated genes in this cell population were enriched for regulators of the immune response (Fig. 2e), with several of these genes found to be upregulated in alveolosphere cultures infected with SARS-CoV-2^36^. In addition to alterations in the cytokine microenvironment, changes in cellular immune populations were also identified in the COPD samples, dysregulation of several genes in inflammatory pathways (*AREG, IL6ST, S100A8/A9, SOCS1*), and high levels of cytotoxic and exhaustion-related genes in CD4 and CD8 T Cells from COPD lungs. Expression of cytotoxic and exhaustion genes was increased compared to controls but similar in IPF and Other-ILD immune cell types. Together, our data suggest that the immune microenvironment at both the molecular and cellular level in the fibrotic and COPD lung is dysregulated and may promote severe infection and poor outcomes in COVID-19.

One limitation of our study is that we focus mainly on the peripheral regions of the lungs, and do not analyze cells in the upper airways or trachea. It is possible that there are significant differences in SARS-CoV-2 entry gene expression between disease and control samples in the more proximal regions of the lungs. Our study is also limited to the expression profiles of patients with CLD without SARS-CoV-2 infection, as samples from patients who are both infected with SARS-CoV-2 and have chronic lung disease are difficult to collect at present. In addition, scRNA-seq is inherently limited to analyses of gene expression which does not always correlate with protein levels. The net effect of many changes in gene expression levels is also difficult to predict and requires determination in experimental models. For example, our analysis demonstrated increased expression of viral entry restriction factors concurrently with an increased viral entry gene score in diseased epithelial cells. Furthermore, scRNA-seq cannot determine spatial relationships which would preclude analysis of cellular behaviors influenced by neighboring cells such as the priming of the viral spike protein by adjacent protease positive cells^82^. Nevertheless, given the inherent limitations in studying human biology, our study highlights crucial areas for future research into the pathogenesis of COVID-19 in patients with CLD including the dysregulation of genes related to viral replication and the innate immune response in epithelial cells, and basal differences in inflammatory cell gene expression programs.

## Methods

### scRNA-seq samples

scRNA-seq data were obtained from published data with samples in the “VUMC/TGen” dataset from Habermann et al.^32^ (GEO accession GSE135893), samples in the “Yale/BWH” dataset came from Adams et al.^31^ (GEO accession number GSE136831), samples in the “Pittsburgh” dataset from Morse et al.^30^ (GEO accession GSE128033) and samples in the “Northwestern” dataset from Reyfman et al.^29^ (GEO accession GSE122960) (Supplementary Tables 1, 2). For specific IRB review of each dataset, please refer to the original paper cited here. In addition, there are 39 unpublished scRNA-seq samples in the “VUMC/TGen” dataset that were collected under Vanderbilt IRB #’s 060165, 171657 and Western IRB # 20181836.

### scRNA-seq data processing

Seurat v4.0 package^83^ was used to process the scRNA-seq data. Specifically, for the Pittsburgh, Northwestern datasets and 39 unpublished samples from the VUMC/TGen, the CellRanger (10X Genomics) output files were read into Seurat using the function *Read10X*, the remaining datasets were already in Seurat format and were loaded using the function *readRDS*. To eliminate low-quality/dying cells or empty droplets, we removed any cells containing fewer than 1000 genes or more than 25% mitochondrial genes. Due to the large size of the joint dataset, we performed *SCTransform*^84^ for normalization and scaling of each dataset separately, split into four major cell populations using known markers: *EPCAM* + (Epithelial), *PECAM1* + *PTPRC* - (Endothelial), *PTPRC* + (Immune) and *EPCAM- PECAM- PTPRC-* (Mesenchymal). Each population from the four datasets was then merged together to generate four merged Seurat objects (Endothelial, Epithelial, Immune and Mesenchymal). Next, each object was SCTransformed with “dataset” being used as batch_var to eliminate batch effects between datasets. Cell clustering was performed using the Seurat function FindNeighbors and FindClusters and cell type annotation was manually performed on each object using known cell-type specific markers (Supplementary Fig. 1^32^). For each cell population, cell type annotation was performed at four levels, ranging from the most general to more granular annotation. Cells expressing more than one cell type specific marker were identified as doublets. After removing doublet cells, all four population datasets were merged to generate the final ILD object containing a total of 611,398 cells and 32 distinct cell types (Supplementary Table 3, Supplementary Fig. 1).

### Integrated analysis of joint dataset

To calculate the percentage of single positive or double positive cells for *ACE2* and other cofactors, we counted the number of cells with >0 transcripts of corresponding genes. For double positive, cells have >0 transcripts of both genes of interest.

To assess the expression profile of SARS-CoV-2 mediators (*ACE2*, *BSG*, *NRP1, HSPA5)*, the corresponding proteases (*TMPRSS2*, *CTSL*, *FURIN*) and other candidate genes involved in SARS-CoV-2 infection in different chronic lung disease subset (COPD, IPF or Other-ILD), we ran the function *FindMarkers* in Seurat package using the negative binomial test. Using Seurat function CellCycleScoring, we calculated the cell cycle state across all epithelial cells to ensure the cell cycle is not a factor contributing to the differential expression analysis (Supplementary Fig. 7). To account for batch effects, we used the parameter “*latent_vars*” to incorporate the four variables (Age, Ethnicity, Smoking status and Dataset) into the negative binomial model. For the binary heatmap, the differential expression analysis was performed between the Disease (including all chronic disease subset) and Control samples. Then, log_2_fold-change was converted into 0 (downregulated in the disease samples) or 1 (upregulated in the disease samples) regardless of the Bonferroni adjusted *p* values. Heatmaps were generated from the adjusted log_2_FC values using the *heatmap.2* function of the *gplots* R package^85^. For the boxplots, count numbers of selected genes were plotted using the *geom_boxplot* and *geom_jitter* function of the *ggplot2* R package^86^.

### Gene module score

To calculate the combined expression of genes, we used the *AddModuleScore* in Seurat v3.1.5 package. SARS-CoV-2 entry gene scores were calculated on SARS-CoV-2 receptors and mediators: *ACE2, BSG (CD147), NRP1, HSPA5(GRP78), TMPRSS2, CTSL, FURIN* and *ADAM17*. Viral entry restriction ISGs: *LY6E, CLEC4D, UBD, ELF1, FAM46C, REC8*^43^. Viral replication inhibition ISGs: *SPATS2L, ZBP1, DNAJC6, IFIT3, RGS22, IFIT1, IFIT5, B4GALT5*^43^. HLA type II score includes *HLA-DRA, HLA-DQA1, HLA-DQA2, HLA-DPA1, HLA-DRB1, HLA-DPB1, HLA-DQB2, HLA-DRB5, HLA-DQB1, HLA-DMA, HLA-DMB*. IFN score includes *ISG15, IFI44, IFI27, CXCL10, RSAD2, IFIT1, IFI44L, CCL8, XAF1, GBP1, IRF7, CEACAM1*. IL6 scores were calculated on six tocilizumab responsive genes: *ARID5A, SOCS3, PIM1, BCL3, BATF, MYC* that are associated with the IL-6 pathway^61^. Cytotoxicity associated genes include *PRF1, GZMH, IFNG, NKG7, KLRG1, PRF1*^61^ and *GNLY, GZMB, GZMK*^87^. Exhaustion gene set: *LAG3, TIGIT, PDCD1, CTLA4, HAVCR2, TOX*^63^, and *PRDM1, MAF*^61^. Significant differences between different groups were calculated using the Tukey_HSD statistic test in the R package *rstatix* with a confidence level of 0.95 (Supplementary Dataset 2).

### Gene correlation analysis

To identify genes that correlate with *ACE2* in the AT2 *ACE2* + cells, we performed Spearman correlation coefficient analysis on the log-transformed and normalized data using the function *cor.test* in the R stats v3.6.1 package with Benjamini-Hochberg corrections for p-adjusted values. Gene ontology analysis for the significant correlated genes (*p* value ≤ 0.03 and 99th percentile rho) was performed with the Bioconductor R package TopGO version 2.42 and the Bioconductor annotation data package *org.Hs.eg.db* version 3.12.0; Kolmogorov–Smirnov (KS) statistic method with TopGO default algorithm weight01 test was used for GO term enrichment test^88^, and Benjamini Hochberg (BH) adjusted *p* values (*q* values or FDR) were computed using the R function *p*.adj.

### Immunohistochemistry of ACE2 and anti-αvβ6 integrin

Formalin-fixed paraffin-embedded histological sections of human lung were cut at 5-microns and dewaxed in xylene prior to rehydration in decreasing concentrations of ethanol. The tissue samples were obtained after informed consent and local ethics approval (South East Scotland SAHSC Bioresource-reference number 06/S1101/41; Brompton Node samples—reference number 15/SC/0101; Papworth Node Samples; non-diseased controls- reference number (Q)GM030404 and Nottingham BRC samples- reference number 08/H0407/1). IHC staining was performed using the Novocastra Novolink™ Polymer Detection Systems kit (Code: RE7280-K, Leica, Biosystems, Newcastle, UK) as previously described^89^. Heat-induced citrate antigen retrieval (pH 6.0) and pepsin antigen retrieval was performed for Rabbit monoclonal ACE2 (ab108252, EPR4435(2) Abcam, UK) and the anti-αvβ6 integrin antibody (6.2A1; Biogen, Cambridge, MA, USA), respectively. Rabbit monoclonal ACE2 (1:400) and anti-αvβ6 integrin (1:3000) was diluted in Leica antibody diluent (RE AR9352, Leica, Biosystems, UK) and incubated with the sections overnight at 4 °C. Novolink DAB substrate buffer plus was used as the chromogen and the slides were counterstained with Novolink haematoxylin for 6 min, dehydrated and cover slipped. A negative control without the application of the primary antibody, and was also used to ensure staining was only related to the presence of the antibody.

The immunohistochemically stained slides were scanned using a ScanScope XT Slide Scanner (Leica Aperio Technologies, Vista, CA, USA) under 20× objective magnification (0.5 µm resolution) using Pannoramic Viewer (3DHISTECH Ltd Budapest, Hungary) slide viewing software. Both the percentage of staining and staining intensity of ACE2 expression in lung sections were individually assessed. For ACE2 quantification, the following scoring system of five high-power fields at X40. per tissue section were used:

The coding was performed prior to scoring/analysis as: **0**- Negative; **1**- 0–≤10%; **2**- 11–≤25%; **3**- ≤26%. Statistical analyses were completed using GraphPad Prism 7.0 (GraphPad Software, San Diego, CA, USA). One-way analysis of variance was used for comparison of more than two datasets and significant differences between diagnosis groups were calculated using the Tukey HSD test.

### ACE2 western blot

Cell protein was isolated using Cell Lysis Buffer (Cell signalling, USA) supplemented with protease inhibitor cocktail (Sigma, USA) and the quantification performed using BCA Protein Assay Kit (Thermofisher Scientific, UK). Western blotting was performed using 4–12%, pre-cast Bis-Tris gradient gels (Thermofisher Scientific, UK) and 25 µg of protein was loaded per lane. Immunoblots were incubated with anti-ACE2 (ab108252; Rabbit monoclonal-Abcam-EPR4435(2)—1:500 dilution of stock antibody) diluted in 5% skim milk/Tris buffered saline with 0.1% Tween-20 for overnight at 4 °C. A loading control of GAPDH was also used to demonstrate protein loading (ab8245; Mouse monoclonal-anti-GAPDH antibody [6C5] at 1:10000 dilution of stock antibody). Following day immunoblots were incubated with an anti-mouse-HRP and anti-rabbit-HRP conjugated secondary antibodies (Dako, USA) at 1:2500 for 1 hr at room temperature. Visualization was performed with Clarity Max™ ECL Substrate (Biorad, UK) on a Licor C-DiGit. For more information on the anti-ACE2 antibody, please refer to the manufacturer’s datasheet here: https://www.abcam.com/ace2-antibody-epr44352-ab108252.html. Two replicates were performed for the western blot.

### Statistical analysis

Tukey Honest Significant Difference (Tukey_HSD) statistical test from the R package *rstatix* with a confidence level of 0.95 was used to test statistical dependence of cells expressing the SARS-CoV-2 mediators among chronic disease subsets. Tukey_HSD test was also used to test significant difference in gene expression module score, quantification of cell types and the ACE2 protein expression quantification. Significant differences in gene expression were the Bonferroni adjusted p-values calculated from the FindMarkers function between Control and Disease groups (COPD, IPF, Other ILD) using the fitted negative binomial model and latent_vars parameters as described above. Significance in gene correlation analysis between ACE2 and other SARS-CoV-2 entry factors (Fig. 3a and Supplementary Fig. 10) was calculated using Anova.

### Reporting summary

Further information on research design is available in the Nature Research Reporting Summary linked to this article.

## Supplementary information

Supplementary Information

Description of Additional Supplementary Files

Supplementary Dataset 1

Supplementary Dataset 2

Supplementary Dataset 3

Supplementary Dataset 4

Reporting Summary

## Data Availability

The majority of the data used in this manuscript are publicly available from published papers: GEO accession “GSE135893”^32^, GEO accession “GSE136831”^31^, GEO accession “GSE128033”^30^ and GEO accession “GSE122960”^29^. The unpublished data from VUMC/TGen (39 samples) are included in the supplementary data (Supplementary Dataset 1) as a count matrix format containing all the genes being used in the manuscript. All other relevant data supporting the key findings of this study are available within the article and its Supplementary Information files or from the corresponding author upon reasonable request. Source data are provided with this paper. A reporting summary for this article is available as a Supplementary Information file. Source data are provided with this paper.

## Source data

Source Data

## Code availability

The code for genomic analyses in this paper is available at https://github.com/tgen/banovichlab/Disease_lung_COVID19_2020/.
