# Supplementary material for: Chronic lung diseases are associated with gene expression programs favoring SARS-CoV-2 entry and severity
Source: Nat Commun. 2021 Jul 14;12:4314. doi: 10.1038/s41467-021-24467-0 (PMC8280215; doi:10.1038/s41467-021-24467-0)
Supplement: Supplementary file 1 — Supplementary Information [file 41467_2021_24467_MOESM1_ESM.pdf]

Chronic lung diseases are associated with gene expression programs favoring SARS-CoV-2 entry and severity

Linh T. Bui[1†], Nichelle I. Winters[2†], Mei-I Chung[1], Chitra Joseph[3], Austin J. Gutierrez[1], Arun C. Habermann[2], Taylor S. Adams[4], Jonas C. Schupp[4], Sergio Poli[5], Lance M. Peter[1], Chase J. Taylor[2], Jessica B. Blackburn[2], Bradley W. Richmond[2,6], Andrew G. Nicholson[7,8], Doris Rassl[9], William A. Wallace[10,11], Ivan O. Rosas[12], R. Gisli Jenkins[3], Naftali Kaminski[4], Jonathan A. Kropski[2,6,13 ††], Nicholas E. Banovich[1††] and the Human Cell Atlas Lung Biological Network*

Online Data Supplement

Supplementary Tables 1-5

Supplementary Dataset 1-4

Supplementary Figures 1-12

SUPPLEMENTARY TABLES

Supplementary Table 1:

a. Summary of sample processing method of the samples used in the study

Dataset/Ref.	Tissue Source	Sampling	Digest Method
Northwestern (Reyfman *et al.*, 2019)	Explanted lung, 1 bronchoscopic biopsy	Peripheral biopsy representative of whole lung	Collagenase D, DNase I-> mechanical mincing
Pittsburgh (Morse *et al.*, 2019)	Explanted lung	Apical and basal biopsy	Collagenase A, DNase->Miltenyi GentleMACS Octodissociator
VUMC/TGen (Habermann *et al.*, 2020)	Explanted lung	Apical and basal peripheral biopsy	Collagenase I, Dispase II -> Miltenyi GentleMACS Octodissociator
Yale/BWH (Adams *et al.*, 2020)	Explanted lung	Apicobasal longitudinal biopsy, exclusion of large airways/blood vessels	Elastase, DNase, liberase->mechanical mincing

b. Number of samples used in the study

Dataset	Control	COPD	IPF	Other ILD
Northwestern	9	0	4	4
Pittsburgh	9	0	8	0
VUMC/TGen	22	7	25	15
Yale/BWH	38	24	45	0
**Total**	**78**	**31**	**82**	**19**

Supplementary Table 2: Demographics of lung donors used for scRNA-seq

	Control	COPD	IPF	Other-ILD
	n=78	n=31	n=82	n=19
Age	44.23 [17-80]	62 [55-73]	64.87 [43-78]	54.95 [37-70]
Ever_smoke	29 (37.2%)	28 (90.3%)	52 (63.4%)	8 (42%)
Gender (Male)	44 (56.4%)	19 (61.3%)	59 (71.9%)	10 (52.6%)
Ethnicity				
European	54 (69.2%)	31 (100%)	60 (73.2%)	12 (63.2%)
African	8 (10.2%)	-	3 (3.7%)	3 (15.8%)
Hispanic	1 (1.3%)	-	4 (4.9%)	-
Asian	2 (2.6%)	-	1 (1.2%)	-
Other/Unknown	13 (16.7%)	-	14 (17%)	4 (21%)

Supplementary Table 3a: Cell proportion for each cell type in each dataset or diagnosis group

Cell type	Cell proportion (%)							
	Dataset				Diagnosis			
	Northwestern	Pittsburg	VUMC/TGen	Yale/BWH	Control	COPD	IPF	Other-ILD
AT1	0.73	0.35	0.80	0.22	0.73	1.28	0.10	0.55
AT2	33.17	2.95	10.95	1.82	16.80	4.65	3.86	11.28
B Cells	0.61	0.51	1.03	2.42	0.75	2.77	2.00	0.79
Basal	1.43	2.30	1.61	0.28	0.18	0.22	1.72	1.63
CD4 T Cells	2.76	4.22	3.92	3.37	2.53	6.83	3.60	2.62
CD8 T Cells	0.03	0.24	5.36	7.53	4.51	11.60	5.14	2.46
cDCs	0.58	0.92	3.42	2.75	1.66	3.50	2.85	3.62
Ciliated Cells	4.84	3.86	10.46	3.39	3.22	5.69	8.85	6.11
Fibroblasts	0.30	4.92	1.59	1.49	0.75	1.27	2.29	1.60
HAS1 High Fibroblasts	0.03	0.46	0.21	0.11	0.12	0.07	0.22	0.16
KRT5-/KRT17+	0.09	0.27	0.79	0.18	0.04	0.17	0.67	0.71
Lymphatic Endothelial Cells	0.31	1.51	0.90	0.64	0.89	0.69	0.66	0.80
Macrophages	44.12	54.31	36.52	67.23	59.05	47.37	49.61	47.25
Mast Cells	0.33	1.25	0.71	0.36	0.34	0.86	0.63	0.45
Mesothelial	0.09	0.09	0.33	0.16	0.06	0.37	0.23	0.38
Monocytes	0.00	0.03	0.52	0.01	0.21	0.15	0.16	0.49
MUC5AC+	0.05	0.29	0.51	0.04	0.09	0.56	0.26	0.20
MUC5B+	1.06	1.93	3.14	0.51	0.36	0.37	2.84	2.30
Myofibroblasts	0.09	1.69	0.38	0.42	0.07	0.15	0.81	0.37
NK Cells	0.04	0.06	0.37	2.73	1.24	2.90	1.33	0.17
pDCs	0.09	0.10	0.26	0.25	0.06	0.45	0.26	0.29
Pericytes	0.05	0.66	0.68	0.26	0.05	1.06	0.43	0.64
Plasma Cells	1.07	0.55	0.45	0.30	0.37	0.88	0.39	0.48
PLIN2+ Fibroblasts	0.01	0.27	0.17	0.16	0.12	0.02	0.25	0.06
PNEC/Ionocytes	0.06	0.01	0.20	0.09	0.05	0.09	0.18	0.19
Proliferating Macrophages	1.45	1.67	0.03	0.24	0.22	0.01	0.52	0.57
Proliferating T Cells	0.11	0.08	0.85	0.16	0.32	0.33	0.41	0.80
SCGB3A2+	2.14	2.03	2.39	0.41	0.51	0.71	2.18	2.15
SCGB3A2+/SCGB1A1+	0.76	0.85	2.60	0.40	0.44	0.83	1.76	2.52
Smooth Muscle Cells	0.24	0.85	0.58	0.15	0.19	0.33	0.48	0.45
Transitional AT2	1.59	0.18	0.42	0.15	0.76	0.19	0.23	0.42
Vascular Endothelial Cells	1.76	10.58	7.86	1.76	3.34	3.63	5.08	7.51
**Total cells**	**65,459**	**35,415**	**241,825**	**268,699**	**184,852**	**80,440**	**265,513**	**80,593**

Supplementary Table 3b: Cell proportion for each cell type among datasets or diagnosis groups

Cell type	Cell proportion (%)							
	Dataset				Diagnosis			
	Northwestern	Pittsburg	VUMC/TGen	Yale/BWH	Control	COPD	IPF	Other-ILD
AT1	15.34	4.02	61.90	18.75	43.57	33.22	8.91	14.31
AT2	40.12	1.93	48.92	9.03	57.37	6.91	18.93	16.80
B Cells	4.17	1.89	25.98	67.96	14.48	23.29	55.61	6.62
Basal	14.59	12.76	60.88	11.76	5.19	2.80	71.47	20.54
CD4 T Cells	8.28	6.85	43.36	41.51	21.39	25.17	43.77	9.67
CD8 T Cells	0.07	0.25	38.92	60.76	25.04	28.03	40.99	5.94
cDCs	2.31	1.99	50.57	45.13	18.74	17.22	46.20	17.83
Ciliated Cells	8.13	3.51	64.98	23.38	15.28	11.76	60.32	12.64
Fibroblasts	2.01	17.80	39.30	40.89	14.09	10.46	62.27	13.19
HAS1 High Fibroblasts	1.82	16.62	51.77	29.79	21.88	5.67	59.78	12.66
KRT5-/KRT17+	2.42	3.71	74.80	19.07	3.16	5.46	69.03	22.35
Lymphatic Endothelial Cells	4.39	11.53	46.98	37.10	35.71	12.09	38.18	14.02
Macrophages	9.11	6.07	27.86	56.97	34.43	12.02	41.55	12.01
Mast Cells	6.45	13.22	51.57	28.76	18.77	20.66	49.78	10.79
Mesothelial	4.65	2.52	59.95	32.88	8.09	22.50	46.30	23.11
Monocytes	0.23	0.84	96.05	2.89	29.48	8.97	31.53	30.02
MUC5AC+	2.04	6.92	83.92	7.12	11.53	30.80	46.54	11.13
MUC5B+	6.69	6.60	73.42	13.29	6.38	2.85	72.87	17.90
Myofibroblasts	2.08	22.23	34.11	41.57	4.71	4.42	79.84	11.02
NK Cells	0.30	0.25	10.81	88.64	27.60	28.13	42.67	1.61
pDCs	4.44	2.58	45.23	47.74	7.46	26.16	49.53	16.85
Pericytes	1.31	8.97	63.16	26.56	3.43	32.68	44.19	19.71
Plasma Cells	25.00	6.94	38.91	29.15	24.14	25.21	36.77	13.88
PLIN2+ Fibroblasts	0.62	9.99	43.81	45.58	23.31	1.46	70.14	5.10
PNEC/Ionocytes	5.10	0.51	62.50	31.89	11.10	9.44	60.20	19.26
Proliferating Macrophages	41.98	26.26	3.23	28.52	18.20	0.44	61.12	20.24
Proliferating T Cells	2.90	1.04	78.99	17.07	22.98	10.39	41.64	24.99
SCGB3A2+	15.51	7.99	64.19	12.31	10.42	6.30	64.08	19.20
SCGB3A2+/SCGB1A1+	6.12	3.70	76.87	13.31	9.88	8.17	57.06	24.89
Smooth Muscle Cells	7.04	13.36	61.73	17.88	15.31	11.81	56.77	16.11
Transitional AT2	41.27	2.53	39.76	16.44	55.80	6.06	24.67	13.47
Vascular Endothelial Cells	4.02	13.09	66.35	16.54	21.54	10.19	47.14	21.14
**Total cells**	**65,459**	**35,415**	**241,825**	**268,699**	**184,852**	**80,440**	**265,513**	**80,593**

Supplementary Table 4: *ACE2+* vs. *ACE2-* CLD AT2 unique genes

Genename	p_val	avg_log2FC	pct.1	pct.2	p_val_adj
SFTPA1	4.03E-09	0.578156076	0.94	0.881	0.000191942
SFTPD	1.56E-08	0.451851465	0.909	0.757	0.000745609
ACSL4	2.19E-08	0.400087955	0.614	0.468	0.001043338
TFPI	2.66E-08	0.494254003	0.705	0.553	0.001265523
ADGRF5	2.96E-08	0.373739604	0.79	0.649	0.001412447
HHIP	4.56E-08	0.588375405	0.636	0.43	0.002172491
LPCAT1	4.83E-08	0.37670078	0.884	0.748	0.002303051
DHCR24	6.43E-08	0.293833821	0.901	0.782	0.003065095
DMBT1	7.60E-08	0.636001537	0.628	0.447	0.003622058
CPB2	1.53E-07	0.292152883	0.352	0.197	0.007275621
PRDX5	3.50E-07	-0.301825135	0.963	0.954	0.016661234
TMEM191C	4.62E-07	0.111689942	0.151	0.066	0.022033042
TSPAN1	4.93E-07	-0.58961901	0.173	0.307	0.023506553
ABCA3	6.65E-07	0.415711847	0.852	0.68	0.031713974
NPC2	8.30E-07	0.28895014	0.986	0.979	0.039565458
CACNA2D2	9.74E-07	0.317392333	0.668	0.485	0.046403494
KCNJ15	9.97E-07	0.300923175	0.628	0.424	0.047529834
CRTAC1	1.04E-06	0.3164135	0.514	0.346	0.04966531
MYL6	1.48E-06	-0.238926251	0.952	0.974	0.070506091
PGC	1.53E-06	0.470976088	0.827	0.685	0.073129647

Supplementary Table 5: Top 20 enriched GO terms of the significant *ACE2* correlated gene set in the CLD AT2 cells using Kolmogorov-Smirnov (KS) statistic test, q-value is the Benjamini-Hochberg corrected p-value

GO.ID	Term	Annotated	Significant	Expected	p_value	q_value
GO:0070125	mitochondrial translational elongation	88	0	0.58	1.20E-12	0
GO:0070126	mitochondrial translational termination	89	0	0.59	5.20E-12	0
GO:0006406	mRNA export from nucleus	111	1	0.73	2.30E-09	0
GO:0006364	rRNA processing	188	1	1.24	3.00E-08	1.00E-04
GO:0043488	regulation of mRNA stability	169	1	1.11	3.40E-08	1.00E-04
GO:0000209	protein polyubiquitination	313	2	2.06	4.30E-08	1.00E-04
GO:0015031	protein transport	1733	7	11.42	6.70E-08	1.00E-04
GO:0042795	snRNA transcription by RNA polymerase II	68	1	0.45	1.90E-07	4.00E-04
GO:0016032	viral process	798	5	5.26	2.60E-07	4.00E-04
GO:0000398	mRNA splicing, via spliceosome	346	0	2.28	2.80E-07	4.00E-04
GO:0016579	protein deubiquitination	254	0	1.67	4.80E-07	7.00E-04
GO:0032981	mitochondrial respiratory chain complex I assembly	59	0	0.39	6.20E-07	8.00E-04
GO:0006120	mitochondrial electron transport, NADH to ubiquinone	47	0	0.31	1.10E-06	0.0013
GO:0006281	DNA repair	520	3	3.43	3.10E-06	0.0032
GO:0016567	protein ubiquitination	767	4	5.05	3.40E-06	0.0032
GO:0006886	intracellular protein transport	1029	4	6.78	3.40E-06	0.0032
GO:0032436	positive regulation of proteasomal ubiquitin-dependent protein catabolic process	84	0	0.55	3.50E-06	0.0032
GO:0090090	negative regulation of canonical Wnt signaling pathway	165	2	1.09	4.20E-06	0.0036
GO:0006368	transcription elongation from RNA polymerase II promoter	80	0	0.53	4.40E-06	0.0036
GO:0006367	transcription initiation from RNA polymerase II promoter	176	1	1.16	6.10E-06	0.0047

SUPPLEMENTARY FIGURES

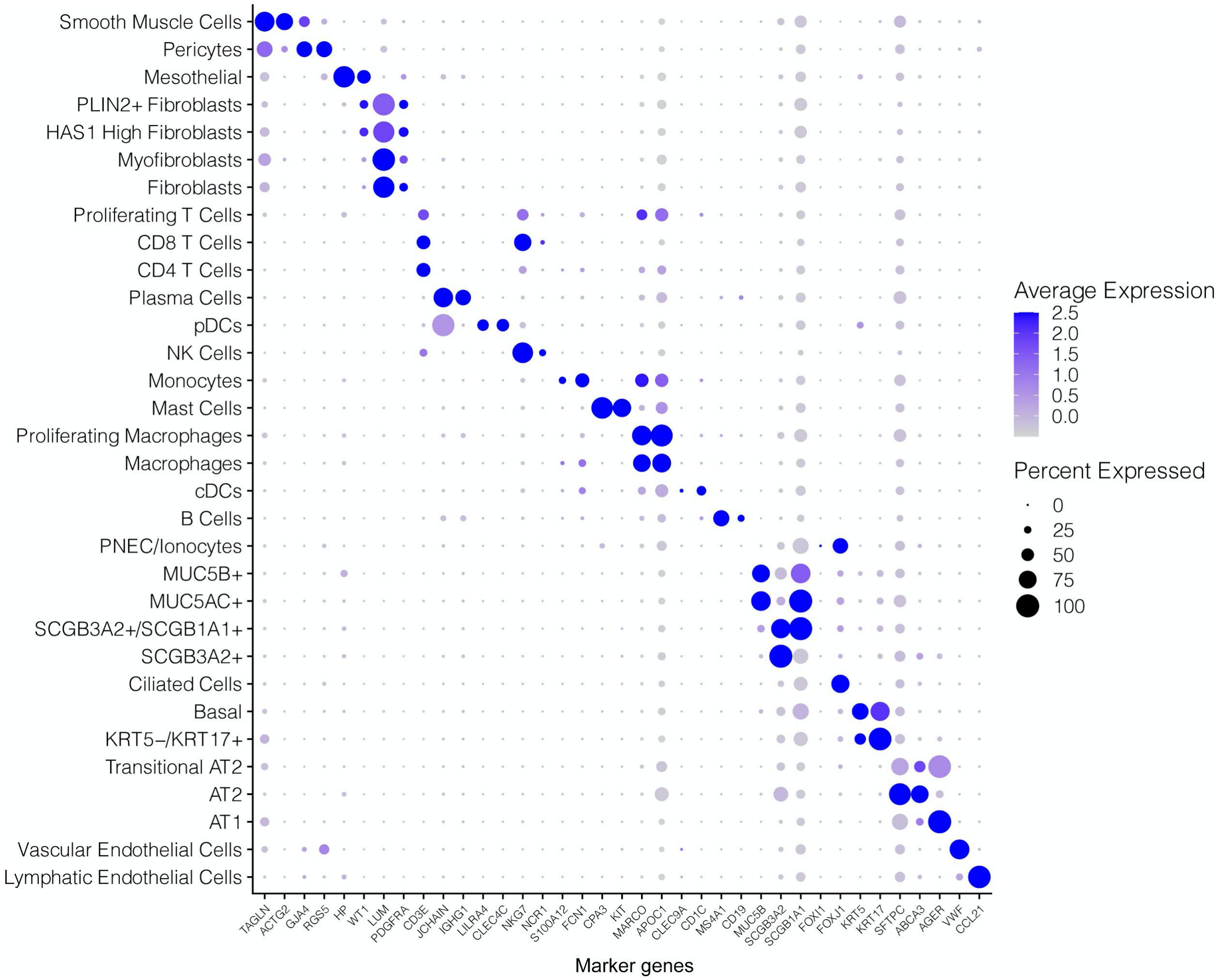

Supplementary Fig 1: Dotplot shows ubiquitous scaled expression of cell type specific marker genes used in cell type annotation of Seurat clusters. The intensity of the dots represents average expression levels of the marker genes in each cell type, while the size of the dots represents the percentage of cells expressing the corresponding marker in each cell type.

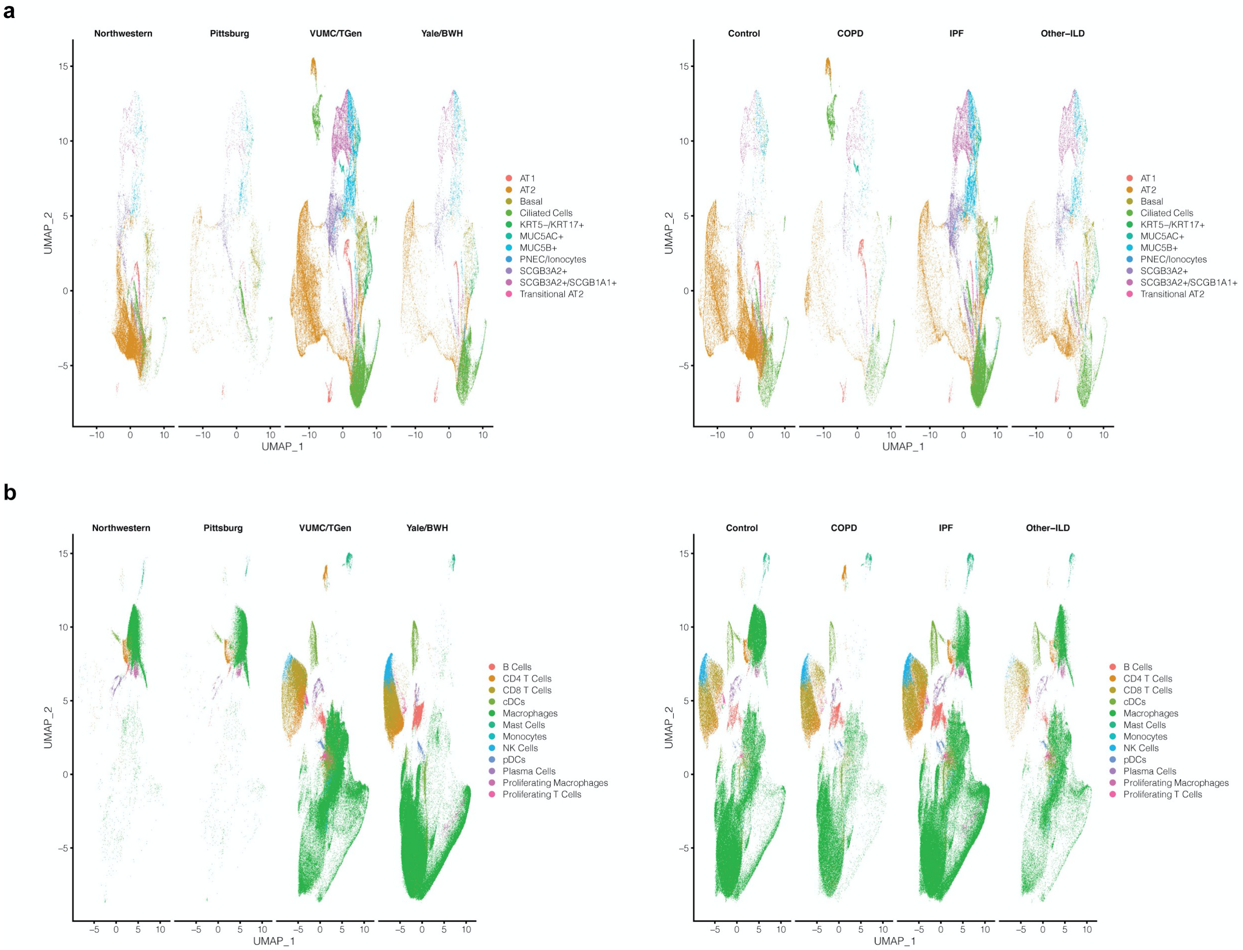

Supplementary Fig 2: UMAP plots display cell type distribution between dataset and diagnosis groups in (**a**) the epithelial and (**b**) the immune cell population.

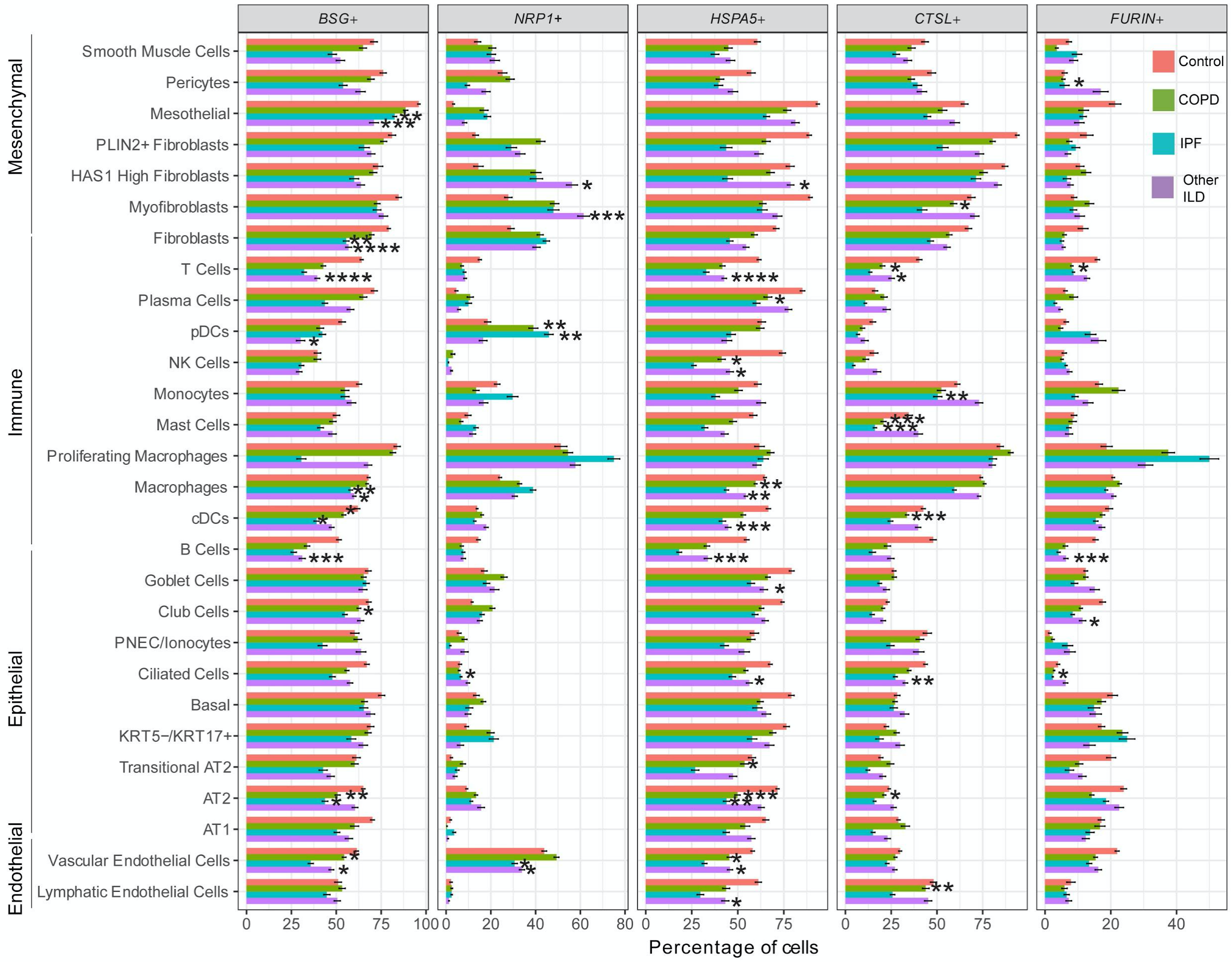

Supplementary Fig 3: Percentage of single positive cells for *BSG* (*CD147*), *NRP1, HSPA5* (*GRP78*), *CTSL* and *FURIN* in different chronic lung disease groups per cell type. Plots were generated with mean values of percentage of cells per individual samples, and bars display the standard errors. Tukey_HSD post-hoc statistical test showed significant differences between each diagnosis subgroup and control samples, p-value < 0.05: *, p-value < 0.01: **, p-value < 0.001: ***, p-value < 0.0001: ****

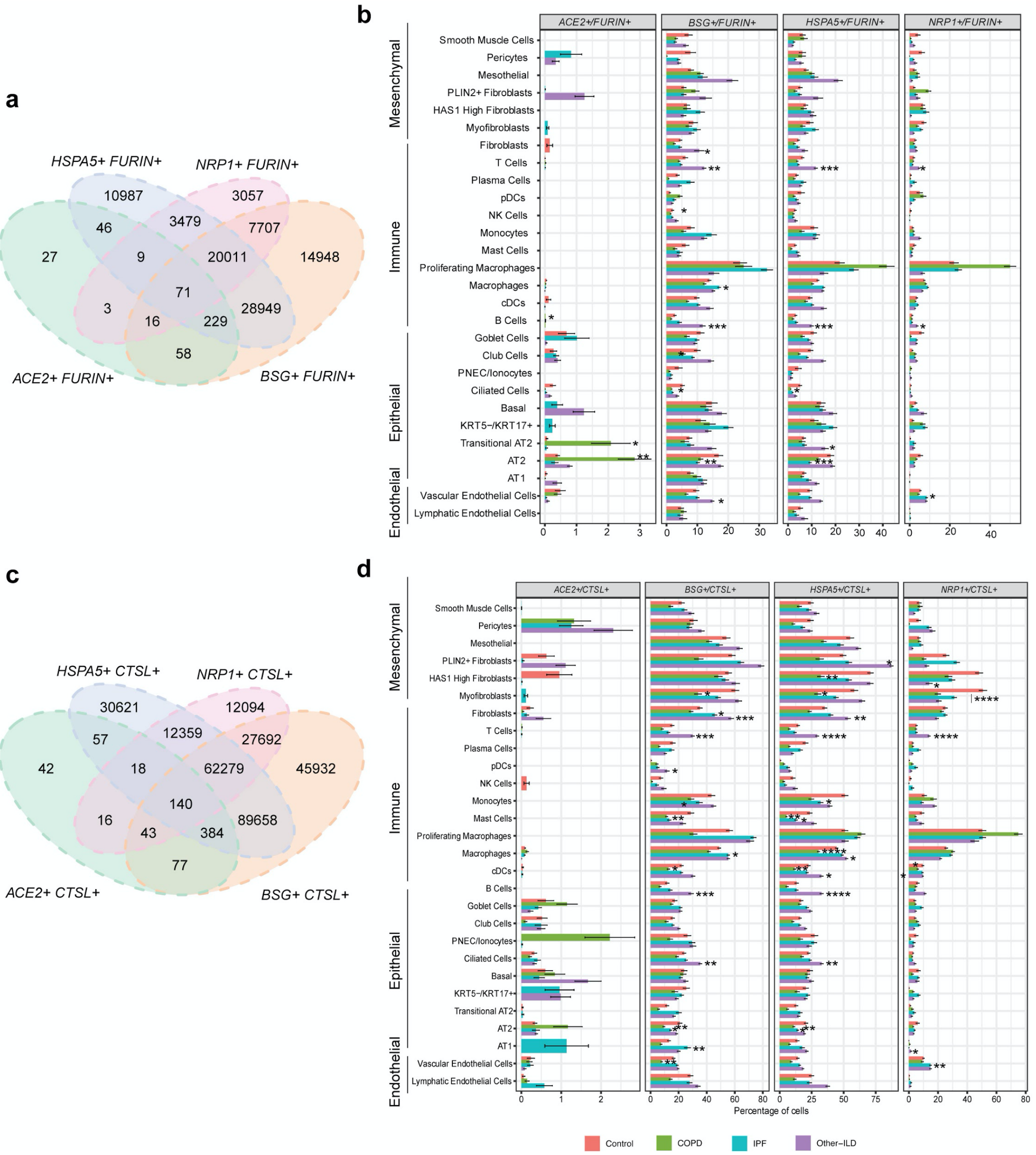

Supplementary Fig 4: Percentage of double positive cells: (**a-b**) *ACE2+ FURIN+*, *BSG+ FURIN+*, *HSPA5+ FURIN+*, *NRP1+ FURIN+* and (**c-d**) *ACE2+ CTSL+*, *BSG+ CTSL+*, *HSPA5+ CTSL+*, *NRP1+ CTSL+* in different chronic lung disease groups per cell type. Plots were generated with mean values of percentage of cells per individual samples, and bars display the standard errors. Tukey_HSD post-hoc statistical test, p-value < 0.05: *, p-value < 0.01: **, p-value < 0.001: ***, p-value < 0.0001: ****

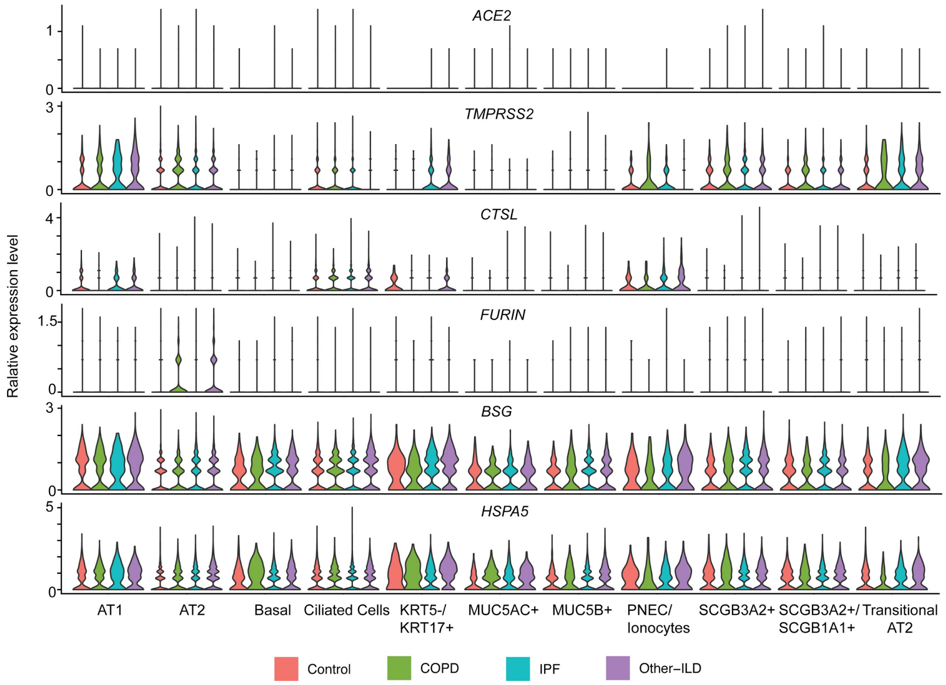

Supplementary Fig 5: Gene expression analysis for SARS-CoV-2 entry mediators in the epithelial cell population.

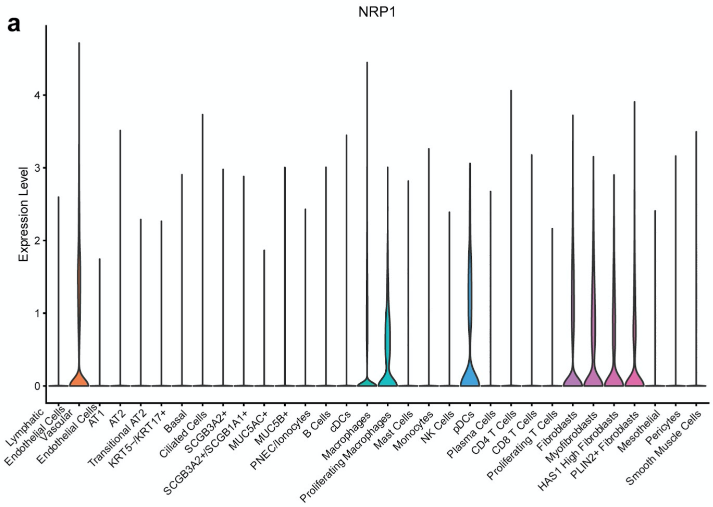

b

CellType	Comparison	p_val	avg_logFC	pct.1	pct.2	p_adj_val
Fibroblasts	COPD_vs_Control	0.92324495	0.09865613	0.44	0.373	1
	IPF_vs_Control	4.67E-06	-0.0923922	0.386	0.373	0.17050783
	Other_vs_Control	0.04199304	-0.2782796	0.302	0.373	1
HAS1 High Fibroblasts	COPD_vs_Control	0.53311716	0.44364169	0.446	0.352	1
	IPF_vs_Control	0.02650629	-0.2368534	0.239	0.352	1
	Other_vs_Control	NA	NA	NA	NA	NA
PLIN2+ Fibroblasts	COPD_vs_Control	0.96932192	-0.0243842	0.357	0.259	1
	IPF_vs_Control	0.64972243	-0.1980843	0.261	0.259	1
	Other_vs_Control	0.99897737	-0.2467766	0.245	0.259	1
Myofibroblasts	COPD_vs_Control	0.00671639	-0.1220705	0.429	0.386	1
	IPF_vs_Control	0.1249954	-0.0760547	0.381	0.386	1
	Other_vs_Control	0.03784285	-0.3365364	0.273	0.386	1
Macrophage	COPD_vs_Control	3.78E-49	0.08897665	0.387	0.327	1.90E-44
	IPF_vs_Control	7.66E-20	-0.1751785	0.321	0.327	3.86E-15
	Other_vs_Control	2.25E-07	-0.4543956	0.229	0.327	0.01132505
Proliferating Macrophage	COPD_vs_Control	0.41975819	-0.0531657	0.4	0.333	1
	IPF_vs_Control	0.45490627	0.14599856	0.401	0.333	1
	Other_vs_Control	0.17588394	-0.1627546	0.265	0.333	1
pDCs	COPD_vs_Control	0.09540542	0.18652572	0.392	0.288	1
	IPF_vs_Control	0.03956713	0.12736751	0.379	0.288	1
	Other_vs_Control	0.99999453	-0.1890941	0.294	0.288	1
Vascular Endothelial	COPD_vs_Control	0.47339912	0.17347171	0.378	0.316	1
	IPF_vs_Control	0.00523269	0.15538032	0.413	0.316	1
	Other_vs_Control	2.77E-12	0.09355556	0.397	0.316	1.00E-07

Supplementary Fig 6: Expression of the putative SARS-CoV-2 receptor, *NRP1*, in the data set. (**a**) Violin plot shows high expression of *NRP1* in some cell types. (**b**) Differential expression analysis of *NRP1* in different disease groups vs. control samples for the cell types having high *NRP1* expression in (**a**). The differential expression analysis was performed using the Seurat FindMarkers function with the four covariates (dataset, age, ethnicity, smoking_status) incorporated into the negative binomial model (as described in the Methods section).

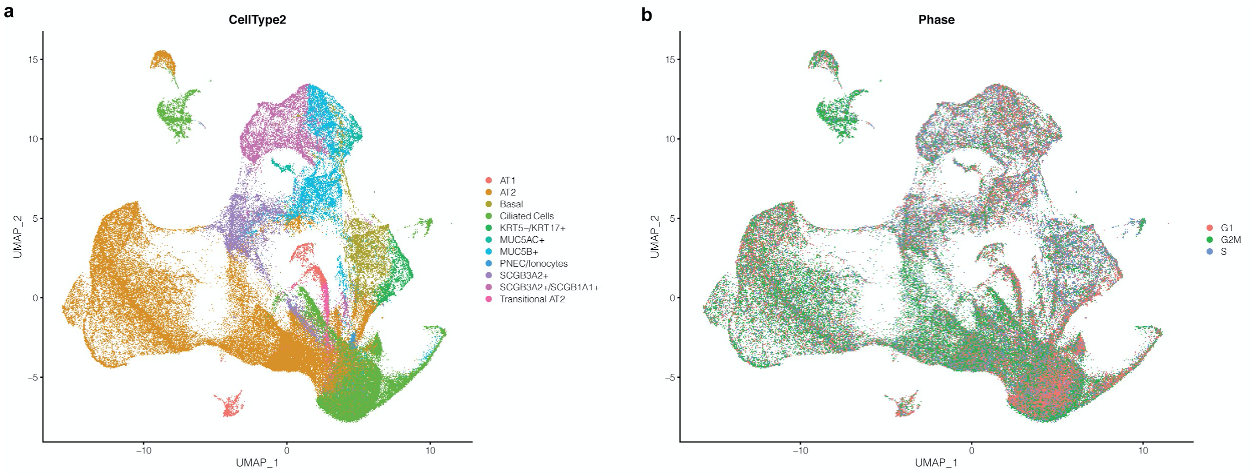

Supplementary Fig 7: UMAP plot shows distribution of cell cycle state across all epithelial cells.

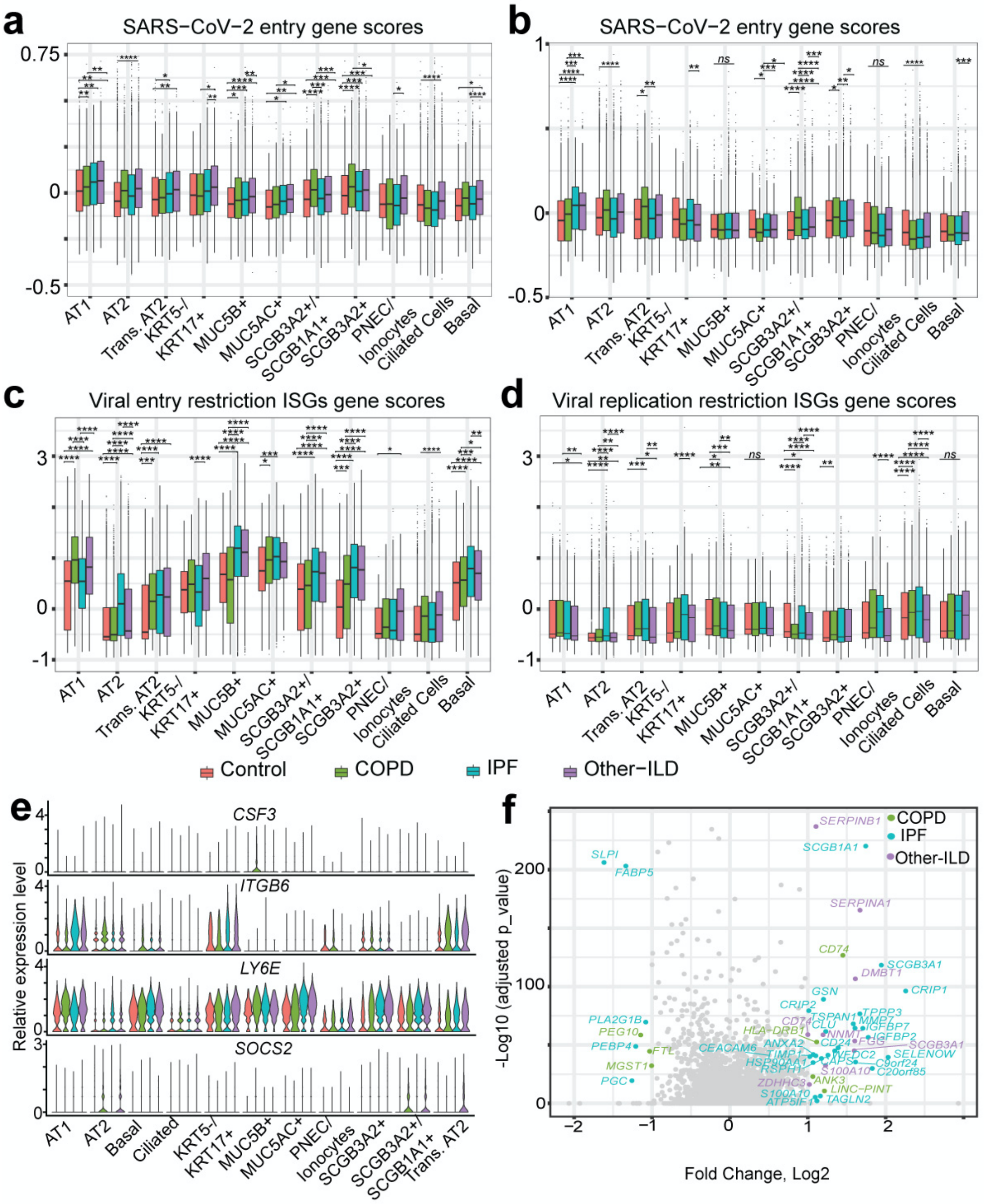

Supplementary Fig 8: SARS-Cov-2 entry genes scores with all outliers included. (**a**) Gene set 1 (*ACE2, BSG, HSPA5, TMPRSS2, CTSL, FURIN, ADAM17*). (**b**) Gene set 2 (*ACE2, TMPRSS2, CTSL, FURIN, ADAM17*). (**c**) Viral entry restriction ISGs gene module scores. (**d**) Viral replication restriction ISGs gene module scores. (**e**) Violin plot shows expression of genes in Figure 2d with all epithelial cell types. (**f**) Volcano plot highlights genes differentially expressed in the AT2 cells between different disease groups and control. Boxes: interquartile range, lower and upper hinges correspond to the first and third quantiles, upper and lower whisker extends from the hinge to the largest values or smallest values of 1.5 x interquartile range (**a**) to (**d**): Tukey_HSD post-hoc statistical test, p-value < 0.05: *, p-value < 0.01: **, p-value < 0.001: ***, p-value < 0.0001: ****.

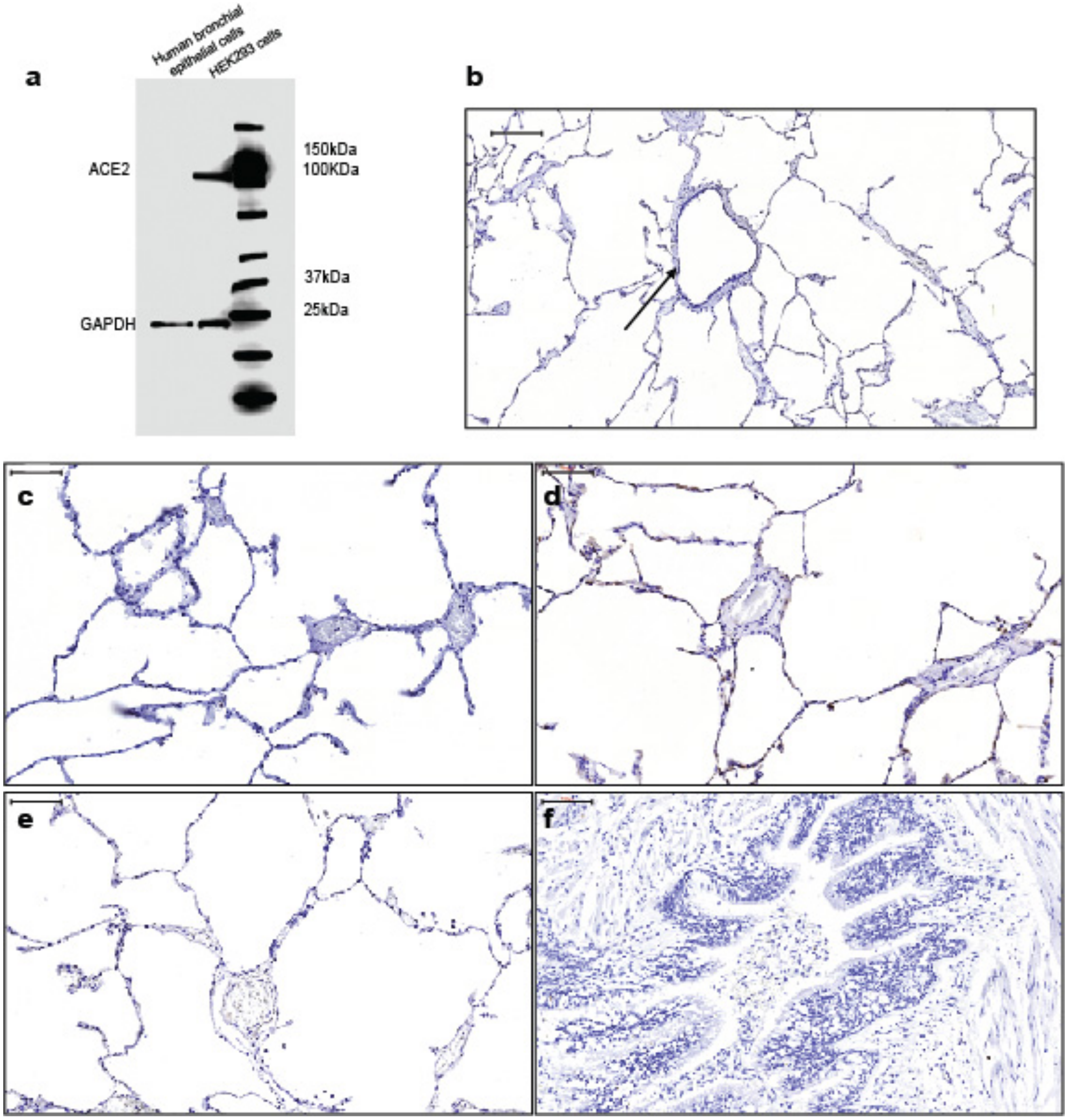

Supplementary Fig 9: Representative sections of control lung sections are shown with each immunomarker. (**a**) Western blotting image showing a single band for ACE2 (ab108252) in the human embryonic kidney HEK293 cells, but not in the human bronchial epithelial cells, at the predicted molecular weight of 100kDa, GAPDH (ab8245) was used as a loading control at 23kDa. ACE2 staining of the small airway (arrow) (**b**) and the alveolar parenchyma (**c**) in the control lung. (**d**) αvβ6 staining in the alveolar parenchyma in the control lung. (**e-f**) Secondary antibody controls for control (**e**) and IPF section (**f**); Scale bars=100 µm. A total of 12 normal lung samples and 62 IPF samples were used for the immunohistochemistry analysis.

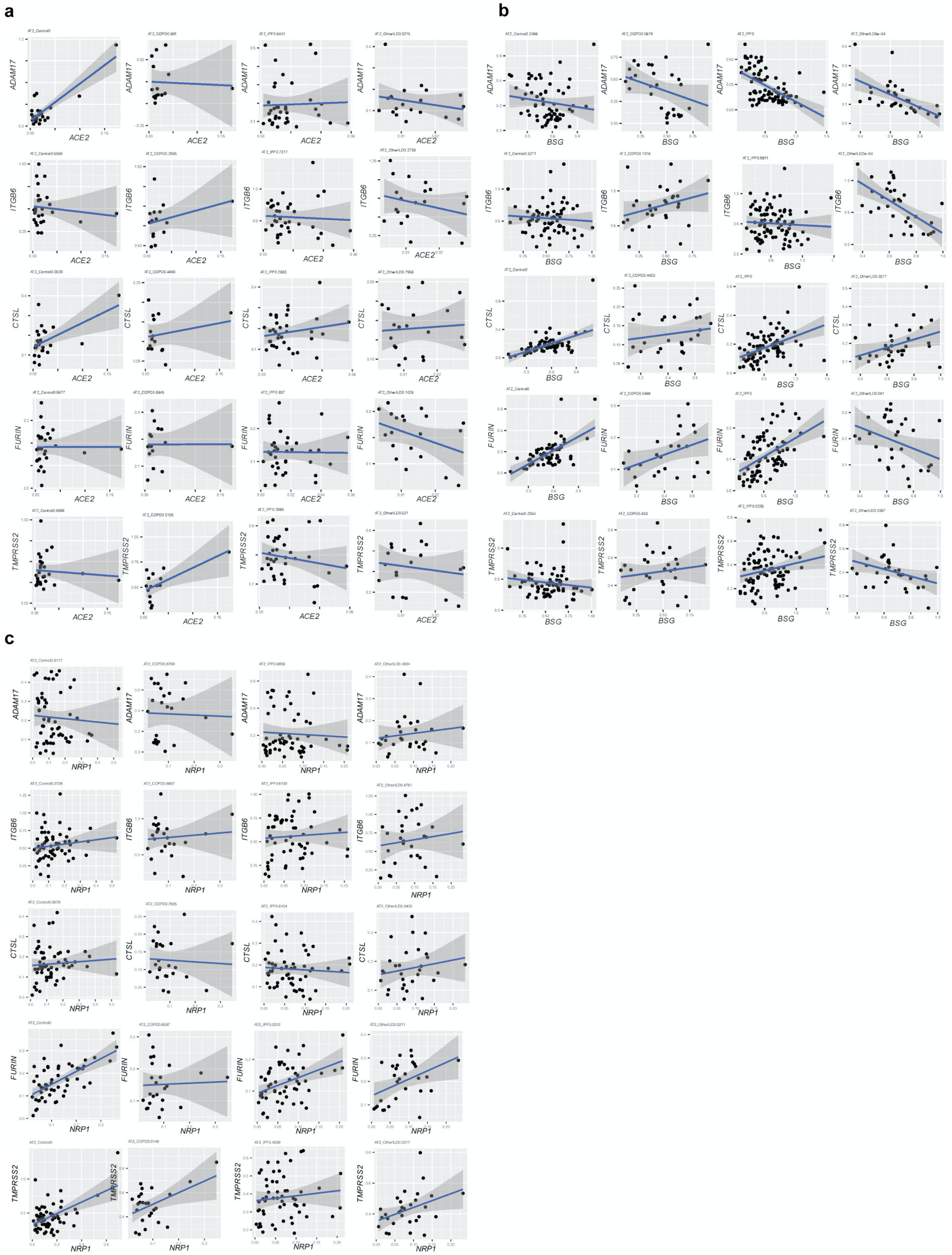

Supplementary Fig 10: Gene expression correlation between SARS-CoV-2 mediators and proteases in the AT2 cell types. (**a**) Gene correlation with *ACE2*. (**b**) Gene correlation with *BSG* (*CD147*). (**c**) Gene correlation with *NRP1*.

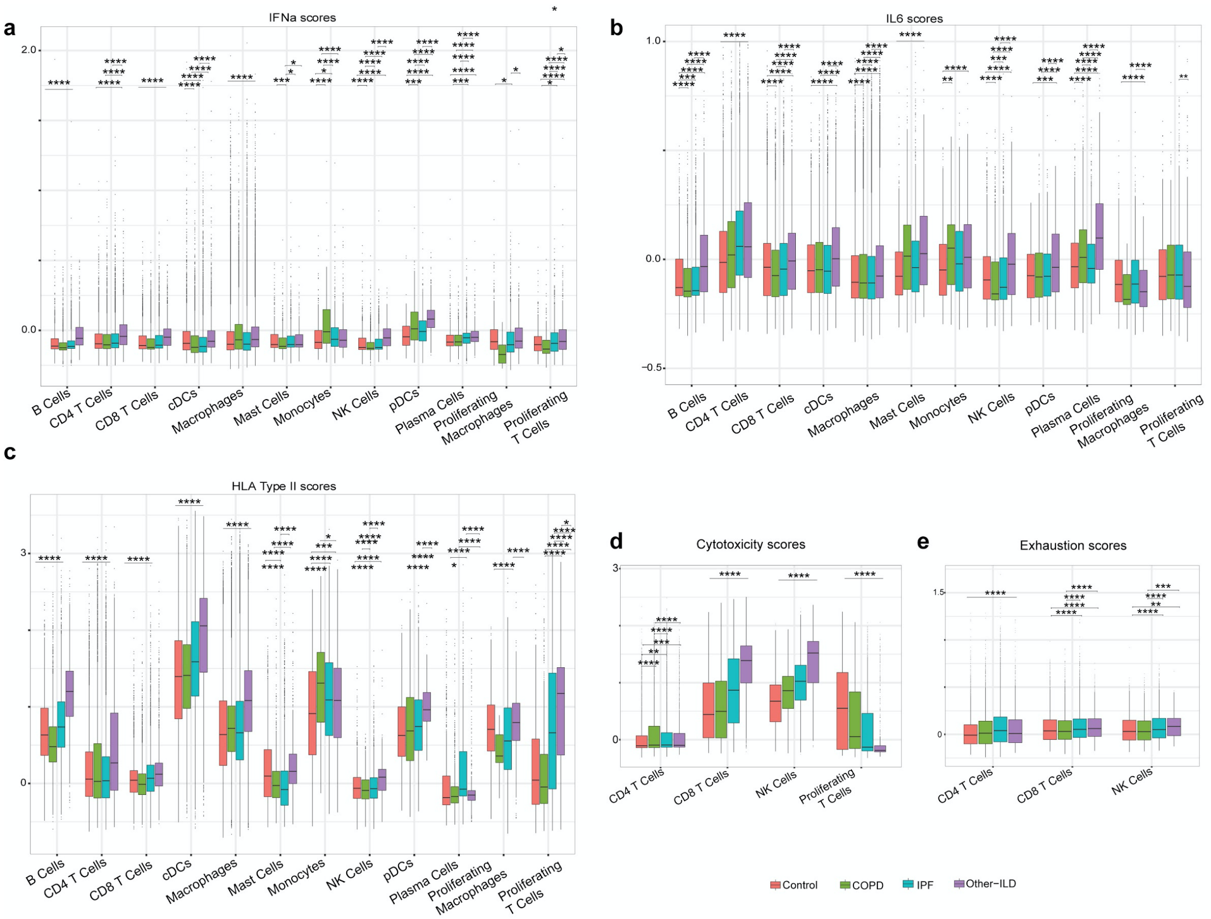

Supplementary Fig 11: Differences in module scores of IFNa (**a**), IL6 (**b**) and HLA type II (**c**) genes in the immune population in different disease groups. (**d-e**) Cytotoxic scores and exhaustion scores in the T cell population in different disease groups. (**b**), (**d**), (**e**) are the same plots as in Fig. 3d-f, but included outliers. Y-axis: gene module score calculated by Seurat AddModuleScore function. Boxes: interquartile range, lower and upper hinges correspond to the first and third quantiles, upper and lower whisker extends from the hinge to the largest values or smallest values of 1.5 x interquartile range. Tukey_HSD post-hoc statistical test, p-value < 0.05: *, p-value < 0.01: **, p-value < 0.001: ***, p-value < 0.0001: ****

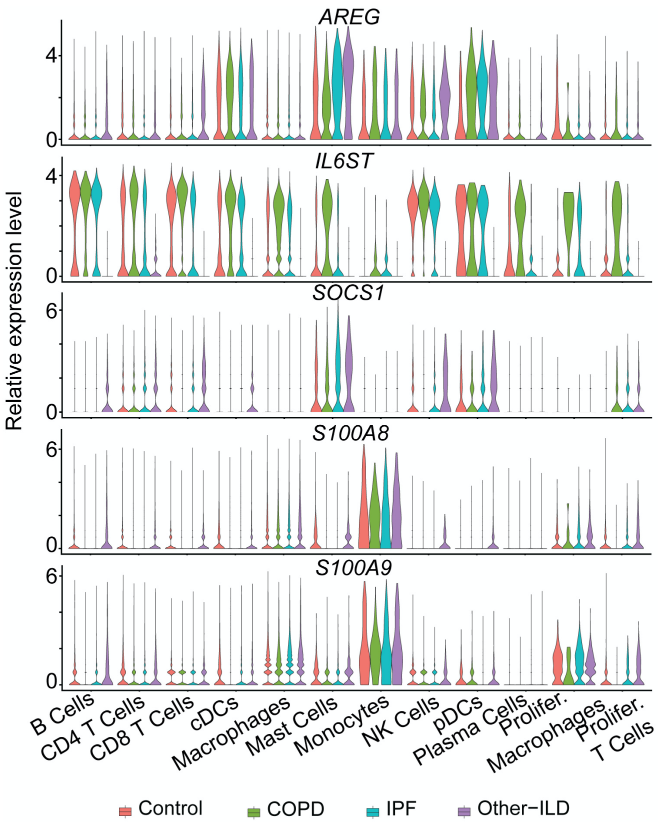

Supplementary Fig 12: Violin plots depict gene expression differences of SARS-CoV-2 immune response genes: *AREG, IL6ST, SOCS1, S100A8* and *S100A9* in all immune cells.
