# Supplementary material for: Chronic lung diseases are associated with gene expression programs favoring SARS-CoV-2 entry and severity
Source: Nat Commun. 2021 Jul 14;12:4314. doi: 10.1038/s41467-021-24467-0 (PMC8280215; doi:10.1038/s41467-021-24467-0)
Supplement: Supplementary file 2 — Description of Additional Supplementary Files [file 41467_2021_24467_MOESM2_ESM.docx]

**Description of Additional Supplementary Files**

File Name: Supplementary Data 1

Description: A matrix contains all raw counts of all the genes used in the manuscript for all unpublished TGen/VUMC samples (39 samples total)

File Name: Supplementary Data 2

Description: An Excel file contains all Tukey_HSD statistical tests used in the manuscript

File Name: Supplementary Data 3

Description: A zip file contains all the results for all differential expression analyses used in Figure 2, Figure 3 and Figure 4

File Name: Supplementary Data 4

Description: An Excel file contains all the results for Spearman correlation coefficient analysis for *ACE2* in AT2 cells (99^th^ quantile of Spearman Rho cutoff)
