# Supplementary material for: Chronic lung diseases are associated with gene expression programs favoring SARS-CoV-2 entry and severity
Source: Nat Commun. 2021 Jul 14;12:4314. doi: 10.1038/s41467-021-24467-0 (PMC8280215; doi:10.1038/s41467-021-24467-0)
Supplement: Supplementary file 7 — Reporting Summary [file 41467_2021_24467_MOESM7_ESM.pdf]

nature research

Corresponding author(s): Nicholas E. Banovich

Last updated by author(s): 2021-17-05

Reporting Summary

Nature Research wishes to improve the reproducibility of the work that we publish. This form provides structure for consistency and transparency in reporting. For further information on Nature Research policies, see our Editorial Policies and the Editorial Policy Checklist.

Statistics

For all statistical analyses, confirm that the following items are present in the figure legend, table legend, main text, or Methods section.

n/a	Confirmed	
[x]	[ ]	The exact sample size (*n*) for each experimental group/condition, given as a discrete number and unit of measurement
[ ]	[x]	A statement on whether measurements were taken from distinct samples or whether the same sample was measured repeatedly
[ ]	[x]	The statistical test(s) used AND whether they are one- or two-sided<br>*Only common tests should be described solely by name; describe more complex techniques in the Methods section.*
[ ]	[x]	A description of all covariates tested
[ ]	[x]	A description of any assumptions or corrections, such as tests of normality and adjustment for multiple comparisons
[ ]	[x]	A full description of the statistical parameters including central tendency (e.g. means) or other basic estimates (e.g. regression coefficient) AND variation (e.g. standard deviation) or associated estimates of uncertainty (e.g. confidence intervals)
[ ]	[x]	For null hypothesis testing, the test statistic (e.g. *F*, *t*, *r*) with confidence intervals, effect sizes, degrees of freedom and *P* value noted<br>*Give P values as exact values whenever suitable.*
[x]	[ ]	For Bayesian analysis, information on the choice of priors and Markov chain Monte Carlo settings
[x]	[ ]	For hierarchical and complex designs, identification of the appropriate level for tests and full reporting of outcomes
[ ]	[x]	Estimates of effect sizes (e.g. Cohen's *d*, Pearson's *r*), indicating how they were calculated

Our web collection on statistics for biologists contains articles on many of the points above.

Software and code

Policy information about availability of computer code

Data collection	Data were collected from existing resources and no software was used for data collection
Data analysis	R packages: Seurat (4.0.1), ggplot2 (3.3.3), upsetR (1.4.0), rstatix (0.7.0), VennDiagram (1.6.0), RColorBrewer (1.1-2), calibrate (1.7.7), topGO (2.42.0), org.Hs.eg.db (3.12.0), RCurl (1.98-1.3), stats (3.6.1)<br><br>10X Genomics CellRanger count (3.1.0)<br><br>The code for genomic analyses in this paper is available at https://github.com/tgen/banovichlab/Disease_lung_COVID19_2020/

For manuscripts utilizing custom algorithms or software that are central to the research but not yet described in published literature, software must be made available to editors and reviewers. We strongly encourage code deposition in a community repository (e.g. GitHub). See the Nature Research guidelines for submitting code & software for further information.

Data

Policy information about availability of data

All manuscripts must include a data availability statement. This statement should provide the following information, where applicable:
- Accession codes, unique identifiers, or web links for publicly available datasets
- A list of figures that have associated raw data
- A description of any restrictions on data availability

The majority of the data used in this manuscript is publicly available from published paper: GEO accession GSE135893 32, GEO accession GSE13683131, GEO accession GSE128033 30 and GEO accession GSE122960 29. The unpublished data from VUMC/TGen (39 samples) are included in the supplementary data (Supplementary Dataset 1) as a count matrix format containing all the genes being used in the manuscript.

Field-specific reporting

Please select the one below that is the best fit for your research. If you are not sure, read the appropriate sections before making your selection.

☒ Life sciences ☐ Behavioural & social sciences ☐ Ecological, evolutionary & environmental sciences

For a reference copy of the document with all sections, see nature.com/documents/nr-reporting-summary-flat.pdf

Life sciences study design

All studies must disclose on these points even when the disclosure is negative.

Sample size	Samples were chosen based on current available single-cell RNA-seq (scRNA-seq) biopsy lung datasets.
Data exclusions	No samples were excluded in the study. We identified doublet cells as cells that co-express several known markers from different cell types and excluded only these cells from the analysis.
Replication	Each dataset contains multiple biological replicates based on specific studies.
Randomization	Randomization is not relevant to the study as the data was not specifically collected for this meta-analysis
Blinding	Blinding is not relevant to the study as the data was not specifically collected for this meta-analysis

Reporting for specific materials, systems and methods

We require information from authors about some types of materials, experimental systems and methods used in many studies. Here, indicate whether each material, system or method listed is relevant to your study. If you are not sure if a list item applies to your research, read the appropriate section before selecting a response.

Materials & experimental systems

n/a	Involved in the study	
☐	☒	Antibodies
☒	☐	Eukaryotic cell lines
☒	☐	Palaeontology and archaeology
☒	☐	Animals and other organisms
☐	☒	Human research participants
☒	☐	Clinical data
☒	☐	Dual use research of concern

Methods

n/a	Involved in the study	
☒	☐	ChIP-seq
☒	☐	Flow cytometry
☒	☐	MRI-based neuroimaging

Antibodies

Antibodies used

For immunohistochemistry:
Rabbit monoclonal ACE2 (ab108252, EPR4435(2) Abcam, UK, 1:400)
Anti-αvβ6 integrin antibody (6.2A1; Biogen, Cambridge, MA, USA, 1:3000)

For Western Blot:
Anti-ACE2 (ab108252; Rabbit monoclonal-Abcam-EPR4435(2) – 1:500 dilution of stock antibody)
Control: mouse monoclonal-anti-GAPDH antibody (ab8245, 1:10000 dilution of stock antibody)

Validation

Validation statements of the listed antibodies are available in the manufacturer's websites:
Rabbit monoclonal ACE2 (ab108252, https://www.abcam.com/ace2-antibody-epr44352-ab108252.html)
Anti-αvβ6 integrin antibody (6.2A1, https://www.freepatentsonline.com/7465449.html)
GAPDH antibody (ab8245, https://www.abcam.com/gapdh-antibody-6c5-loading-control-ab8245.html)

Human research participants

Policy information about studies involving human research participants

Population characteristics	Provided in Supplementary Table 1
Recruitment	N/A (the data was not specifically collected for this meta-analysis)
Ethics oversight	scRNA-seq data were obtained from published data with samples in the “VUMC/TGen” dataset from Habermann et. al. (2020) (GEO accession GSE135893), samples in the “Yale/BWH” dataset came from Adams et. al. (2020) (GEO accession number GSE136831), samples in the “Pittsburgh” dataset from Morse et. al. (2019) (GEO accession GSE128033) and samples in the “Northwestern” dataset from Reyfman et al. (2019) (GEO accession GSE122960) (Supplementary Table 1, 2). For specific IRB review of each dataset, please refer to the original paper cited here. Additionally, there are 39 unpublished scRNA-seq samples in the “VUMC/TGen” dataset that were collected under Vanderbilt IRB #'s 060165, 171657 and Western IRB # 20181836

Note that full information on the approval of the study protocol must also be provided in the manuscript.
